# From viral infection to emission: host barriers shaping airborne transmission

**DOI:** 10.1128/jvi.00753-26

**Published:** 2026-09-28

**Authors:** Patricia Romanos Martinez, Maximilian Rohde, Mohammad Yeasin, Saikat Basu, Christian Sieben, Julia R. Port

**Affiliations:** 1 Laboratory of Transmission Immunology, Helmholtz Centre for Infection Research 28336, Braunschweig, Germany; 2 Nanoscale Infection Biology Group, Helmholtz Centre for Infection Research 28336, Braunschweig, Germany; 3 Department of Mechanical Engineering, South Dakota State University 2019, Brookings, South Dakota, USA; 4 Institute of Genetics, Technische Universität Braunschweig 26527, Braunschweig, Germany; 5 Institute for Virology, Hannover Medical School 9177, Hannover, Germany; New York University Department of Microbiology, New York, New York, USA

**Keywords:** transmission, airborne, aerosol, mucus, respiratory viruses, IRPs

## Abstract

Coughing, speaking, and even quiet breathing can release virus-laden particles into the air, triggering transmission events that spread through households and communities. Understanding why some respiratory viruses transmit so efficiently, and under which biological or environmental conditions this occurs, is central to anticipating future outbreaks. In this review, we outline the biological and physical factors that shape respiratory virus transmission, highlighting how the site of replication, mechanisms of droplet generation, and environmental conditions influence airborne spread. We examine how viral traits such as receptor usage, tissue tropism, and innate immune evasion intersect with airway physiology to determine the size, composition, and infectiousness of exhaled particles. We lastly draw attention to key gaps in current knowledge, particularly regarding the host and environmental factors that govern both the release and uptake of virions. Filling these gaps will allow us to more accurately assess transmission risk and implement targeted approaches to reduce spread.

## INTRODUCTION

Respiratory viruses that infect the lower respiratory tract, including influenza A viruses (IAVs), respiratory syncytial virus (RSV), and coronaviruses, remain a major global health burden. Recent estimates indicate that RSV causes approximately 33 million lower respiratory tract infections, and about 100,000 deaths annually in children under 5 years (1), seasonal influenza accounts for 290,000–650,000 respiratory deaths each year worldwide (2), and severe acute respiratory syndrome (SARS)-CoV-2 has caused more than 7 million reported deaths globally (3, 4). These pathogens remain major drivers of severe respiratory disease, particularly in young children, older adults, and immunocompromised individuals (5, 6). Beyond the substantial annual economic toll of recurring influenza (estimated at over US$11 billion in combined direct medical and indirect costs in the United States alone in 2018), the COVID-19 pandemic underscored how respiratory virus pandemics can destabilize societies and economies worldwide, with its indirect costs estimated at 10.5% of global gross domestic product (7–10).

How do respiratory viruses successfully breach the host barriers? What factors determine whether an exposure leads to onwards transmission, or whether an outbreak escalates into a pandemic? These fundamental questions remain unanswered, rooted in the multifaceted complexity of the transmission process itself. In this review, we explore three stages of transmission: inhalation, exhalation, and transit in the air, with specific attention to how the physicochemical properties of respiratory fluids affect viral stability, how environmental conditions shape airborne persistence, and how host factors influence viral shedding. By combining existing knowledge and insights from the fields of virology, immunology, and aerosol physics, we aim to holistically understand the known determinants of successful airborne transmission, identify crucial knowledge gaps, and suggest urgently needed, but not yet realized, research trajectories increasing pandemic resilience.

## INHALE

### Airflow and particulate deposition

Many respiratory viruses, including IAV, RSV, and SARS-CoV-2, readily establish infection in the upper or lower respiratory tract (URT, LRT). According to current terminology proposed by the World Health Organization, transmission occurs through the transfer of infectious respiratory particles (IRPs) generated during e.g., breathing, speaking, coughing, or sneezing. These particles can reach a susceptible host through three main pathways. First, airborne transmission (inhalation) occurs when IRPs remain suspended in air or are transported by airflows and are subsequently inhaled into the respiratory tract. Second, direct deposition occurs when IRPs follow a short-range trajectory and deposit directly onto exposed facial mucosa, including the mouth, nose, or eyes. Finally, contact transmission occurs when infectious respiratory secretions are transferred either directly between individuals or indirectly via contaminated surfaces, followed by self-inoculation of mucosal surfaces. These pathways are not mutually exclusive, and their relative contribution varies depending on the virus, the host, and environmental conditions (11). IRPs span approximately 0.01-1000µm in diameter. Historically, respiratory particles were divided into “droplets” and “aerosols,” with a 5-μm threshold becoming widely used in medical and infection-control literature through its use as a proxy for respiratory tract deposition, rather than as a strict boundary in aerosol behavior. However, this binary classification is now considered oversimplified. IRP behavior is better understood as a continuum governed by size, airflow, evaporation dynamics, and environmental conditions (12–17). For a historical account of aerobiology research related to IRPs and the evolving definitions therein, the reader may peruse (18).

The anatomical site of deposition is primarily determined by the particle sizes, the inhaled airflow patterns, and the subject-specific geometrical features of the airspace. In general, larger IRPs (e.g., with aerodynamic diameters ≳10 µm) predominantly deposit in the URT (the nasal cavity, nasopharynx, and oropharynx) via inertial impaction and sedimentation (19–22). In contrast, smaller particles, prompted by their low inertia, largely follow the inhaled airflow streamlines in which they are embedded and demonstrate greater penetration levels to the LRT, including terminal airways and alveoli, especially during deep inhalation or mouth breathing. As an additional nuance, airflow during inhalation can also generate shear forces capable of detaching microdroplets from the mucosal lining through viscoelastic fragmentation, potentially transporting virus-containing material deeper into the lung (22–25). Regional deposition has been characterized using complementary approaches, including human inhalation studies, experimental aerosol models, airway replicas, and *in vivo* or *ex vivo* exposure systems (26, 27). Advances in high-fidelity computational fluid dynamics (CFD), informed and validated by these experimental data, now enable quantitative predictions of particle transport and deposition throughout anatomically realistic airways. Together, these approaches provide a mechanistic framework linking inhaled particle size to regional deposition at infection-prone sites and the likelihood of infection establishment (28, 29) (see also “Infectious dose,” below). However, along with deposited pathogen loads at the infection-prone tissue sites, whether an actual infection originates will depend on interactions in both the milieu separating the air from the cell layer and interactions between virus and cell, including the immune response dynamics.

### The mucus milieu

Upon deposition, the inhaled droplet merges with the airway surface liquid. Airway mucus is a hydrated viscoelastic gel composed mainly of large secreted mucins (~2.5 MDa), particularly MUC5AC and MUC5B, together with smaller amounts of DNA, lipids, salts, and globular proteins that modulate its rheological and adhesive properties (30). These components organize into an intricate polymer network that maintains epithelial hydration while trapping inhaled particles and pathogens. The airway surface liquid can be conceptually divided into two layers: the superficial mucus gel and the underlying periciliary layer (PCL), a low-viscosity region that allows cilia to beat freely. Together, they form what has been termed the “gel-on-brush” model, in which the cilia project through the PCL to propel the overlying gel (31). Within this structure, mucin polymers assemble through disulfide-bonded cysteine-rich domains, creating extended, dynamic networks with pore sizes ranging from roughly 100 to 500nm.

**Fig 1:**
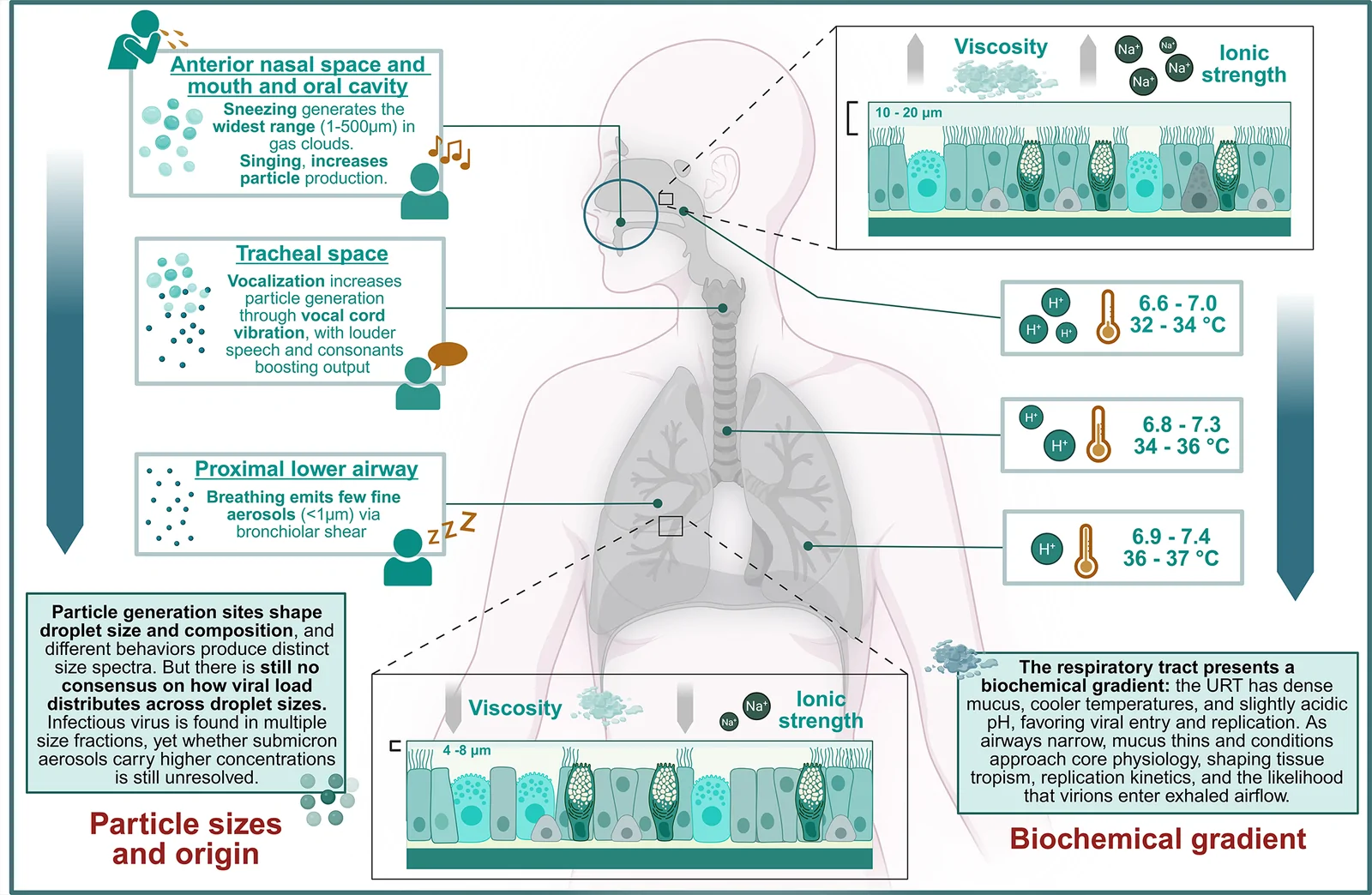
Anatomical and biochemical determinants of respiratory particle generation and deposition.

Mucus composition and architecture are not uniform along the respiratory tract (Fig. 1) (32). Regional differences in airway surface hydration, ionic composition, and pH generate distinct physicochemical microenvironments along the respiratory tract. In humans, nasal and lower-airway surface liquids differ in Na^+^, Cl^−^, and K^+^ concentrations, while the nasal surface is generally more acidic than the lower airways, although absolute values vary with anatomical location and measurement methodology (33, 34). In the nasal passages, mucus is dense, rich in glycans, and highly viscous, providing an effective molecular sieve and contributing to air humidification. The tracheobronchial mucus is more hydrated and optimized for transport, forming a continuous flow that clears inhaled material toward the pharynx. The distal bronchioles and alveolar regions are covered by a progressively thinner lining fluid, with the alveolar surface characterized by a surfactant-rich rather than mucus-dominated layer (35). The pH of airway mucus is close to neutrality (~URT = 6.8, LRT = 7.2), but it can shift toward more acidic or alkaline values during infection or inflammation, affecting mucin conformation, charge distribution, and gel viscoelasticity (30, 36, 37). Even modest changes in ionic strength or divalent cation concentration can markedly alter the degree of mucin cross-linking, tightening or loosening the network, and thereby modifying its permeability.

Viruses interact with airway mucus through a combination of steric exclusion, electrostatic interactions, and specific glycan-mediated binding. Mucin glycans can function both as structural components of the gel network and as decoy receptors that capture incoming particles. In model systems, particles with neutral or hydrophilic surfaces diffuse more readily through mucin gels than those with exposed charges, highlighting the importance of surface chemistry in determining mobility within mucus (38). Similar principles appear to apply to viruses. IAVs attach to terminal sialylated glycans through hemagglutinin (HA) but avoid permanent trapping by enzymatic cleavage of these receptors via neuraminidase (NA). The balance between receptor binding and receptor-destroying activity determines whether virions remain immobilized within mucus or progress toward the epithelial surface (39–42).

Comparable strategies are observed for several other respiratory viruses. Some betacoronaviruses, including human coronaviruses OC43 and HKU1, encode a hemagglutinin-esterase (HE) protein that acts as a receptor-destroying enzyme capable of cleaving O-acetylated sialic acids, thereby regulating virion attachment and release from mucosal glycans (43–45). Evolutionary studies indicated that the spike (S) protein and HE co-evolved to maintain an optimal balance between receptor binding and detachment, analogous to the HA-NA functional balance in influenza viruses. Interestingly, human-adapted OC43 and HKU1 appear to have partially reduced HE receptor-binding activity compared with bovine or other animal coronavirus counterparts, suggesting evolutionary adaptation to the environment of the human airway mucosa (43). Parainfluenza virus NA activity can cleave sialic acid residues within nasal mucus glycoproteins, altering the structure of the mucin gel and potentially facilitating viral penetration through the mucus network. Experimental studies using bovine nasal secretions demonstrated that NA activity disrupts local gel architecture by releasing negatively charged sialic acid residues, resulting in localized collapse and restructuring of the mucin network. These observations suggest that viral enzymatic activities can directly modify mucus microstructure to promote particle mobility (46, 47). In contrast, viruses lacking receptor-destroying enzymes may experience stronger mucoadhesive interactions and reduced mobility within the airway mucus layer. Experimental evidence from single-particle tracking supports this concept: pseudorabies virus particles diffused substantially more slowly in porcine tracheal mucus than in aqueous environments, indicating strong trapping by mucus through a combination of electrostatic and adhesive interactions (48). Notably, surface modification of viral particles with neutral hydrophilic polymers significantly increases their mobility, highlighting the importance of particle surface chemistry in determining diffusion through mucus. These principles are likely applicable across respiratory viruses. For coronaviruses, such as SARS-CoV-2, the extensive glycan shielding of the spike protein may reduce nonspecific electrostatic interactions with mucins, facilitating partial diffusion through the mucus layer (49). RSV lacks a neuraminidase capable of cleaving sialylated glycans, and its mobility within airway mucus may therefore depend more strongly on local physicochemical conditions. However, direct experimental measurements of RSV diffusion in healthy human airway mucus remain limited. During infection, host responses, such as neutrophil extracellular trap (NET) formation, can trap RSV particles within dense DNA-rich mucus plugs, which may restrict viral spread but also contribute to airway obstruction in severe disease (50).

Differences in airway and mucus-associated glycan repertoires among host species are increasingly recognized as contributors to viral tropism and may form part of interspecies transmission barriers, particularly for glycan-binding respiratory viruses, such as IAV (39, 49, 51–53). Dehydration, inflammation, or oxidative stress can shrink pore size and increase cross-linking, thus restricting diffusion (41, 42, 54, 55). Conversely, disruption of disulfide bonds within mucin polymers can enlarge pores and enhance viral mobility (56).

**Fig 2:**
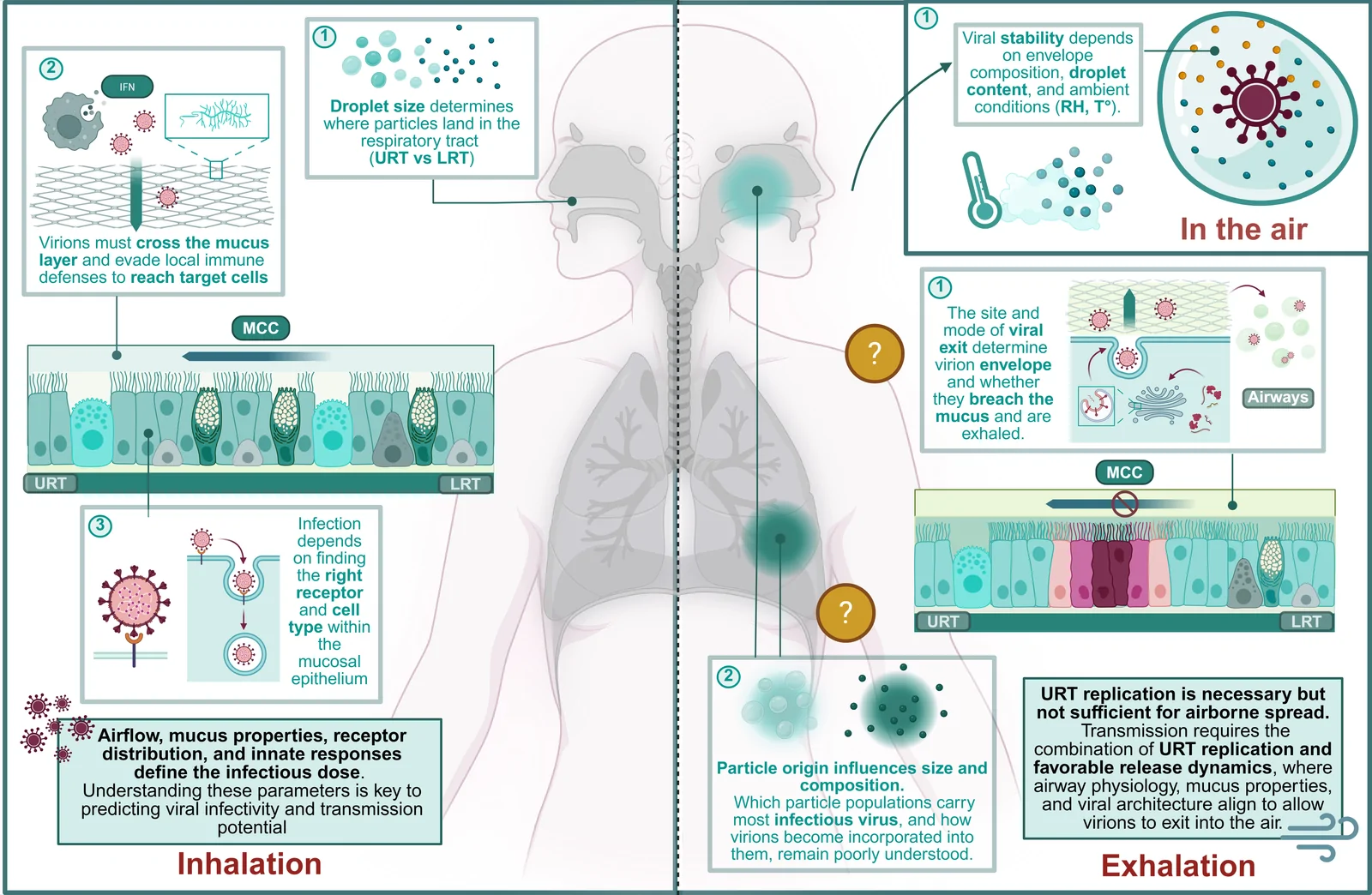
From deposition to aerosolization: biological and physical determinants of airborne transmission.

Once a virion reaches the epithelial surface, its fate depends on the dynamic interplay between mucus transport and receptor accessibility (Fig. 2). The continuous motion of mucus, driven by coordinated ciliary beating, constitutes the mucociliary clearance (MCC) mechanism. While MCC is primarily an antiviral defense, recent work indicates that once infection is established, it can also influence the spatial pattern of viral spread. For example, in differentiated human airway epithelial cultures, live imaging showed that early SARS-CoV-2 infection can expand in the direction of mucus transport, indicating that intact ciliary motion and mucus flow may facilitate local spread along the epithelial surface (57). As infection progresses, however, SARS-CoV-2 caused loss and dysfunction of motile cilia, which impairs MCC and may favor retention of infectious material at the airway surface and potentially facilitate expansion of infection (58). Similar disruption of mucociliary function has been observed during RSV infection, where early ciliary dyskinesia, cilia loss, and epithelial damage reduced effective mucus transport and may promote viral persistence at the airway surface (59). For IAV, short-term exposure was reported to transiently increase ciliary beat frequency and cilia-driven flow in airway epithelium (60), but this response is currently interpreted as an early host-defense mechanism. Evidence that it promotes viral spread deeper into the lower respiratory tract remains limited. The concept of “mucus surfing” has been proposed to describe this phenomenon (31, 61–63). Temperature, pH, and exposure to pollutants, such as cigarette smoke, all modulate ciliary beat frequency, mucus secretion, and oxidative status, ranging from temperature-dependent increases in ciliary activity to long-term smoke-induced ciliary loss and dysfunction; thereby influencing the balance between defense and dissemination (64–67).

### Receptor landscape and viral tropism

Once a virion crosses the mucus barrier, successful infection depends on a precise match between viral attachment and entry factors, and the host’s epithelial landscape. This defines the concept of viral tropism, which not only dictates which cells are infected first but also determines both the efficiency of transmission and the severity of disease that follows (68–70).

Respiratory viruses show distinct spatial tropisms along the airway, but receptor distribution alone does not fully determine where replication occurs. IAVs, human coronaviruses, and SARS-CoV-2 all replicate efficiently in the URT (71–73); however, this reflects not only receptor availability but also the local temperature, mucus environment, and entry pathway preferences. This can be exemplified with SARS-CoV-1, as its receptor, angiotensin converting enzyme (ACE)2, is present in both upper and lower airway epithelia, yet the virus was predominantly associated with lower respiratory tract replication and alveolar damage (74–76), illustrating that receptor presence is necessary but not sufficient for productive infection. Measles virus (MeV) further illustrates this. After inhalation, MeV is thought to initiate infection predominantly in CD150-positive macrophages and dendritic cells. Following amplification and systemic dissemination through CD150-positive immune cells, MeV subsequently infects nectin-4-positive airway epithelial cells from the basolateral side, facilitating epithelial spread and apical shedding (77–79).

The efficiency of virus-receptor binding is modulated by temperature and structural factors, as the respiratory tract exhibits a thermal gradient from the cooler nasal passages (33°C) to the warmer lower airways (37°C) (Fig. 1). Several studies show that SARS-CoV-2 spike-mediated entry and replication are enhanced at temperatures typical of the URT (~33°C), whereas SARS-CoV-1 and Middle East respiratory syndrome (MERS)-CoV appear less adapted to these cooler airway conditions and exhibit more efficient spike activity at temperatures closer to 37°C (72, 80–85).

Similarly, human-adapted influenza viruses show preferred binding to α2,6-linked sialic acid receptors, which are abundant in the URT, and encode PB1 polymerase genes that lose function at febrile temperatures, unlike avian-adapted influenza PB1 variants which remain efficient at ~40°C and their HA spike proteins preferably attach to α2,3-linked sialic acids enriched in the LRT (86, 87).

Target cells also differ in their glycocalyx composition, local pH, and exposure to surfactant components, all of which modulate viral attachment and fusion. For coronaviruses, spike activation is often a multistep process in which different host proteases act at different stages. In SARS-CoV-2 and other coronaviruses, furin commonly cleaves the spike during biosynthesis or egress in producer cells, whereas TMPRSS2 activates spike at the target-cell surface during entry; accordingly, the local availability of activating proteases can help shape tissue tropism and entry route (88–90). SARS-CoV-2 variants exemplify how evolutionary changes in spike processing and entry pathways can alter viral tropism. Early Omicron subvariants (e.g., BA.1) displayed reduced efficiency of TMPRSS2-mediated membrane fusion relative to Delta and showed greater compatibility with endosomal entry routes, a shift associated with reduced fusogenicity and altered lower-airway tropism (91, 92). However, subsequent work in primary airway models and *in vivo* systems has shown that transmembrane protease serine 2 (TMPRSS2) remains an important determinant of infection for many Omicron lineages, and that later subvariants such as BA.5 and BA.2.86-descended variants partially recovered TMPRSS2-dependent entry phenotypes (93, 94). These lineage-specific shifts in entry pathway usage illustrate how relatively small changes in spike processing or protease dependence can reshape anatomical tropism and, consequently, patterns of viral transmission and pathogenesis.

### Early innate immune responses

Innate immunity influences transmission primarily by determining the timing and magnitude of viral replication in the airways. Many highly transmissible respiratory viruses, including SARS-CoV-2, influenza viruses, and RSV, shed most efficiently early in infection (95–98), often before the adaptive immunity is engaged (99). During this period, type I and III interferons (IFNs) provide the main antiviral barrier. Viral infection of airway epithelial cells is first sensed by pattern-recognition receptors that detect, among other things, viral RNA, triggering IFN production. Secreted type I and III IFNs then act in an autocrine and paracrine manner to induce interferon-stimulated genes (ISGs), establishing an antiviral state that restricts viral replication and spread (100). In the respiratory tract, type III IFN (IFN-λ) appears particularly important at epithelial barrier surfaces, where it provides localized antiviral protection with less systemic inflammatory cost. In nasal epithelial cells, IFN-λ is the predominant interferon induced by several respiratory viruses, including RSV, whereas type I IFNs may be less prominent at this site (101). Experimental transmission studies further support this concept: in mouse models, loss of IFN-λ signaling increased IAV nasal shedding and transmission to contacts, whereas prophylactic IFN-λ treatment conferred durable antiviral protection in the URT and limited onward spread (102). Respiratory viruses therefore encode potent IFN antagonists that delay this response long enough to permit high-titer shedding. Well-characterized examples include, IAV NS1 blocks RIG-I-mediated sensing (103), and MeV P, C, and V proteins inhibit STAT1 signaling (104), contributing to the prolonged presymptomatic phase that facilitates MeV transmission (105–107). SARS-CoV-2 uses multiple proteins, including Nsp1, ORF6, and ORF9b, to suppress host translation, preventing STAT1/STAT2 nuclear translocation, and inhibiting MAVS signaling (108–111). Similar strategies are also evident in other respiratory viruses, even if less characterized. Rhinoviruses are associated with impaired type I IFN responses in bronchial epithelial cells, and 3C protease can interfere with apoptosis-related antiviral pathways through cleavage of RIPK1, thereby promoting replication (112, 113). RSV likewise appears to exploit weaknesses in epithelial innate defense while also driving inflammatory remodeling of the airway environment by triggering exaggerated neutrophil extracellular trap formation (50, 114, 115). This can further influence transmissibility by altering the physical properties of airway secretions: NETs increase mucus viscoelasticity (116) and can trap viral particles (117, 118), potentially modifying how virions move through airway fluids and how droplets form during coughing or breathing. These biological effects intersect with exposure modeling frameworks, in which infectious-dose predictions (28, 29), can be refined by incorporating an innate-immunity correction factor into Ω (119), allowing the corrected parameter, evaluated at the critical exposure duration T, to approximate pathogen-specific infectious doses (120). Although this review focuses on the naïve host, it is worth noting that adaptive immunity, particularly mucosal antibodies, can further limit viral spread by neutralizing virions at their sites of release and entry.

### Infectious dose

The infectious dose refers to the minimum number of virions required to establish a productive infection in the host, rather than merely reaching the respiratory tract. However, this threshold cannot be understood as a fixed value, as it emerges from a series of sequential and interdependent processes that occur from the moment particles are inhaled to the initiation of infection at the cellular level. These processes, as discussed previously, include the availability of susceptible target cells, the frequency with which infectious virus-containing droplets are deposited in the airways, the ability of virions to penetrate the mucus and resist mucociliary clearance, the local availability of host factors that facilitate viral attachment, entry, and replication, and the absence or attenuation of effective innate immune responses that would otherwise abort infection at an early stage. As a result, the infectious dose reflects not only viral properties but also the efficiency of these intermediate steps, which can vary substantially depending on the route and conditions of exposure.

In practice, quantitative estimates of infectious dose in humans remain limited. Classical human challenge studies provide some of the most direct insights, but these typically rely on clinical endpoints and therefore approximate the dose required to cause detectable disease, rather than infection per se. This distinction is important, as infection and disease severity are not necessarily equivalent, and asymptomatic or mild infections may still contribute to transmission. Within this framework, differences in the route of exposure further illustrate how infectious dose is shaped by upstream processes. Aerosol inoculation has been associated with more severe disease outcomes for several viruses, including IAV, adenoviruses, and SARS-CoV-2. In the case of IAV, early volunteer studies demonstrated that aerosol exposure can result in infection at very low doses, in some cases as low as one tissue culture infectious dose (TCID)_50_, whereas substantially higher inocula are required for intranasal infection (121–123). Similarly, adenovirus type 4 causes more severe disease when delivered via aerosol compared with intranasal inoculation (124). In contrast, rhinovirus and coxsackievirus require higher doses to establish disease when delivered as aerosols than when administered as nasal droplets, supporting the view that the nasal mucosa represents the primary site of infection for these pathogens (121, 124). Experimental studies with rhinovirus demonstrate that infection is readily achieved through self-inoculation of the nasal or conjunctival mucosa via contaminated hands, whereas oral exposure is ineffective. In contrast, transmission between individuals is inefficient following short-duration exposure in the absence of direct contact with fresh nasal secretions but becomes highly reproducible under prolonged close-contact conditions (125–128). By contrast, the high transmissibility of MeV is well established epidemiologically, but the minimum infectious dose in humans has not been directly quantified in modern challenge experiments. Instead, its high transmissibility is inferred from epidemiological observations and airborne transmission patterns documented during outbreaks (129, 130).

Given these limitations, recent approaches have sought to estimate infectious dose through integrative modeling. For emerging viruses with unknown infectious doses, *in silico* modeling of inhaled transport combined with biological features of the pathogen (comprising information on the infection-prone tissue regions specific to the virus) and related epidemiological observations (particularly comprising transmission metrics such as exposure durations during superspreading events) can provide mechanistic estimates of the pathogen’s infectious dose. For instance, for SARS-CoV-2, computational studies have shown that IRPs primarily in the ~2.5–19-µm-range deposit at the infective tissue sites along the nasopharynx, which bears a high propensity of ACE2 and a relatively thin defensive mucus layer (28, 131). Computing the viral load depositing at the nasopharynx during the critical exposure duration, namely 2.5 h, reported in a known superspreading event (132), the infectious dose was predicted to be approximately 300 virions (28). The prediction on the typical hazardous particle sizes was later supported by measurements in hospital wards housing infected patients (133–135). Quantitatively, the computational approach can define the deposited viral load over an exposure duration T as Ω = V × ξ × T, where ξ is the pathogen concentration in the inhaled IRPs and V represents the CFD-predicted deposition volume at the intra-airway infective tissue sites per unit exposure duration. Here, ξ is typically estimated from saliva or sputum samples collected from diseased subjects (29, 136–138), corrected for environmental dehydration of droplets before inhalation (139). To note, however, that intra-airway fluid film breakup comprises an important mode of exhaled particle generation. Particulates formed from film breakup may carry a higher concentration of microorganisms than in the bulk fluid (which is currently the basis for estimating *ξ*). Therefore, the current modeling approach with the pathogen concentrations derived from nasal/throat swabs can underestimate exposure. Additionally, any credible CFD that aims to eventually replace experimental work must capture not only deposited pathogen loads at infection-prone tissue sites but also the processes at the air-tissue interface, virus-cell interactions, and the resulting immune response dynamics that jointly determine whether infection is established.

For a summary of this section, see Box 1.

Box 1. **Inhale**

Airflow and particle deposition

- Particle size and airflow determine deposition site, defining which airway regions and cell types are initially exposed.

Mucus milieu

- Particle size and airflow determine deposition site, defining which airway regions and cell types are initially exposed.

Receptor and cell type

- Receptor determines viral entry, but the capacity of infected epithelial cells and tissue to support productive replication ultimately shapes tissue tropism, transmission, and disease severity.

Innate immune response

- Early innate responses constrain initial replication and shape the timing and magnitude of peak viral load, influencing the transmission window.

- Infection probability reflects the fraction of inhaled virions that overcome physical, cellular, and immune barriers to establish replication.

## EXHALE

The next step is to follow the reverse journey: how newly formed virions leave the host and re-enter the air through the process of exhalation.

### Mechanisms of viral exit

The directionality of viral egress is a fundamental determinant of whether infection results primarily in localized infection or deeper pathogenic spread. The route of exit (budding, vesicular trafficking, or syncytial spread) determines the microenvironment that surrounds the emerging virion and ultimately its stability and infectivity once outside the cell. Epithelial cells of the respiratory tract are highly polarized, with a distinct apical surface facing the airway lumen and a basolateral domain directed toward the interstitium (140). This polarity governs the direction of progeny release (141–144). Most viruses that infect mucosal surfaces exit through the apical side of epithelial cells (141–143), ensuring their release into the airway lumen where they can be expelled in respiratory secretions and particles (142, 143).

Experimental studies in polarized airway cultures support this distinction. SARS-CoV-1, SARS-CoV-2, and IAVs replicate and exit predominantly through the apical surface of polarized airway epithelial cells, producing viral titers several orders of magnitude higher apically than basolaterally (12, 142, 145–147). This apical bias provides a mechanistic basis for their capacity to reach the airway lumen; however, for SARS-CoV-1, much of this replication occurs in the lower respiratory tract, where limited access to the upper airways constrains shedding and helps explain its relatively poor transmissibility despite apical egress. In contrast, basolateral release is associated with within-host propagation and more severe disease, as exemplified by MERS-CoV (144), which can release from both surfaces but relies heavily on basolateral spread, contributing to deep lung pathology and minimal human-to-human transmission. MeV may exit basolaterally to infect immune cells through nectin-4 and CD150 receptors, consistent with its systemic dissemination, but direct confirmation in airway epithelium is limited (148).

The process of viral egress is tightly coupled to host cell polarity and trafficking machinery. Viruses hijack the same cellular pathways that deliver proteins and vesicles to the plasma membrane, redirecting them to sites of assembly and budding, exploiting pre-existing lipid microdomains enriched in cholesterol and sphingolipids. These lipid rafts serve as organizing platforms that concentrate viral envelope proteins and ensure that the newly formed virion acquires the correct lipid and protein composition. In coronaviruses, progeny particles assemble within the endoplasmic-reticulum-Golgi intermediate compartment and are transported to the cell surface in post-Golgi vesicles, fusing with the apical membrane through an exocytic route (145, 149). RSV, MeV, or SARS-CoV-2 can also spread via cell-cell fusion, forming syncytia that bypass direct exposure to the lumen while maintaining replication within the epithelium (79, 150, 151).

For enveloped viruses, the host membrane defines a critical component of viral identity. Because apical and basolateral membranes have distinct lipid compositions, virions acquire an apical or basolateral lipid signature at the site of assembly and budding (152). Variations in membrane cholesterol, phospholipid content, and lipid-raft organization across cell types and airway regions can alter the physical properties of the viral envelope, its curvature, fluidity, and resistance to desiccation. These factors, in turn, influence how virions interact with mucus, how long they remain infectious in the environment, and how efficiently they fuse with subsequent target cells (153, 154). Some viruses specifically assemble in cholesterol-rich domains that increase envelope rigidity and stability, improving survival in expelled droplets, while others rely on more fluid membranes that facilitate fusion but reduce environmental persistence. Host metabolic or inflammatory states may alter epithelial lipids, subtly changing virion “signatures” and potentially influencing tropism, egress polarity, and transmissibility.

Viruses released apically from superficial ciliated cells are readily incorporated into droplets generated by airflow or coughing. In contrast, virions produced in deeper submucosal or glandular cells may remain trapped within mucus or local tissues, reducing transmission potential but possibly contributing to persistence and tissue damage. The biogenesis of a virion’s exit therefore dictates not only its release route but also its fate in the extracellular environment.

### Anatomical sources of exhaled virus

Infections confined primarily to the LRT are typically associated with increased disease severity but limited onward spread. Human challenge studies showed that deeper respiratory deposition of coxsackievirus, adenovirus, or IAV produced more pronounced disease at relatively low inoculated doses (122, 124). Across respiratory viruses, pathogens with predominant LRT tropism, including SARS-CoV-1, MERS-CoV, and poorly transmissible avian influenza viruses, generally cause more severe disease while exhibiting comparatively limited human-to-human transmission. This pattern may reflect differences in tissue damage, accessibility of infected cells to the airway lumen, and the efficiency with which infectious progeny are incorporated into exhaled particles, together with the limited contribution of alveolar regions to outward airflow and particle generation (121, 122, 155–158).

By contrast, early and robust URT replication is consistently associated with airborne transmission, although whether its involvement is strictly required or determines transmission efficiency remains debated. IAV and SARS-CoV-2 provide some of the strongest evidence for this association. But, while both viruses achieve robust replication in human nasal and upper airway epithelium, mechanistic proof of the link URT replication and airborne release has only been exemplified in animal studies. In ferret models, only IAV replicating in the URT transmits via airborne particles, even when comparable viral loads are present deeper in the respiratory tract (159). Mutations enhancing affinity for α2,6-linked sialic acids, particularly in the mucin-rich soft palate, further increase shedding efficiency and support cross-species adaptation (160). More recently, genetically barcoded SARS-CoV-2 has provided complementary evidence in Syrian hamsters: the relative abundance of individual viral barcodes in the nasal turbinates, but not in the trachea or lungs, predicted their probability of transmission to a subsequent host, identifying the URT as the predominant source of transmitted virus in this model (161).

However, URT replication alone is not sufficient to ensure efficient airborne transmission. RSV and parainfluenza viruses replicate efficiently in the nasal epithelium, yet are transmitted predominantly through direct contact and large-droplet spread, with comparatively limited evidence for sustained airborne transmission (150, 162–164). These findings, while requiring cautious extrapolation to humans, support the hypothesis that efficient transmission depends not simply on viral burden but on when and where productive replication occurs relative to airway physiology and the release of infectious virions, with additional factors, such as receptor usage, cell-exit routes, mucus interactions, infection kinetics, and post-release virion stability, likely shaping the deterministic effects of URT replication.

### The mucus milieu

The site of particle generation largely determines size and composition (Fig. 1 and 2 ). Respiratory particles originate from several distinct regions, including the lower respiratory tract, vocal folds, pharyngeal and laryngotracheal surfaces, anterior nasal airspace, and oral cavity.

In the deep lung, cyclic airway closure and reopening during normal breathing generates submicron particles through fluid film rupture, typically producing relatively few fine particles (<1 µm) (165, 166). As airflow moves upward, additional mechanisms come into play. Phonation and speech induce vibration of the vocal folds and rupture of mucus films along laryngeal and pharyngeal surfaces, yielding particles that span the submicron to low-micron range (167, 168). Articulation during speech, particularly plosive consonants, further fragments mucosal films and creates jet-like flows capable of transporting particles over distances exceeding one meter (169, 170). Singing amplifies these processes through sustained vocal fold vibration, markedly increasing particle output, especially at higher volumes (171). More forceful expiratory events produce broader size distributions: coughing releases particles across approximately 1–100 µm at velocities of 10–22 m/s, typically peaking at 5–15 µm with thousands of particles emitted per event (165, 172), while sneezing generates the widest spectrum (1–500 µm) within turbulent multiphase gas clouds that can carry particles several meters from the source (173–175). Viscoelastic fragmentation of the mucosalivary film, especially during violent expiratory events, has also been recently shown to emit smaller particles with aerodynamic diameters ≲ 10 µm (176).

Across experimental systems, viral RNA and infectious virus are most consistently detected in small respiratory particles, spanning IRPs in the approximate range of ~0.1–20 µm. For SARS-CoV-2, the best-characterized virus to date, evidence for this association comes largely from hospital-based sampling studies, which repeatedly report viral RNA and infectious virus in these smaller particle fractions (131, 133, 177, 178). Despite this apparent enrichment, the field has not yet reached a consensus on how viral load is quantitatively distributed across droplet sizes in infected individuals, and whether submicron IRPs systematically carry higher concentrations of virions than larger droplets remain unresolved.

### Timing of viral shedding across disease course

The timing of viral release during the course of infection determines not only the likelihood of transmission but also the epidemiological profile of the disease. In respiratory infections, peak infectious shedding often occurs before or near the onset of symptoms, when viral replication in the upper airways is maximal and epithelial integrity remains largely intact (98, 179, 180). At this stage, the mucociliary clearance and normal airflow promote continuous shedding of virus-laden droplets. For viruses, such as IAV and SARS-CoV-2, the period of highest contagiousness precedes or coincides with the first signs of illness, which explains their efficient community spread and short serial intervals (98, 179–182). Although coughing and sneezing can generate large bursts of IRPs, they often occur after the peak of infectious shedding, when much of the exhaled virus is already non-viable. In contrast, presymptomatic individuals exhale virus continuously through normal respiration and speech, contributing disproportionately to transmission chains. This temporal mismatch, between perceived illness and actual infectiousness, complicates control measures and underlies the silent spread of pandemic respiratory viruses.

As infection progresses, epithelial damage and inflammation begin to reshape the environment of viral release. Destruction of ciliated cells, accumulation of mucus and cellular debris, and altered ion transport all reduce mucociliary clearance (57–59). Consequently, viral particles may persist in the lower tract or become trapped within viscous sputum rather than being expelled, reflecting a shift from active shedding to intracellular or trapped viral material.

The duration of viral shedding varies. IAV and SARS-CoV-2 display early, short-lived peaks, whereas RSV and parainfluenza tend to maintain lower but prolonged shedding, consistent with their transmission via close contact (183, 184).

For a summary of this section, see Box 2.

Box 2. **Exhale**

Mechanisms of viral exit

- Egress polarity and host-membrane composition shape which virions reach the airway surface and how transmissible they become.

Anatomical sources of exhaled virus

- Replication site and transmission mode are linked but separable: LRT viruses can reach the URT, yet still fail to appear in exhaled IRPs.

Timing of viral shedding

- High shedding can peak early, when mucociliary transport and airflow remain intact and before epithelial damage restricts release.

## IN THE AIR

Having traced how viruses reach the airway lumen, the next step is to understand their fate beyond the host, how expelled particles evolve, travel, and retain infectivity in the surrounding air.

### Droplet transformation after exhalation

When a droplet leaves the airways, it transitions from a warm, fully saturated environment (~34°C–37°C; ~100% relative humidity [RH]) to cooler, drier ambient air. This abrupt shift initiates rapid evaporation of the droplet. Early aerosol studies described viral decay using biphasic kinetic models that distinguish an initial rapid-loss phase from a slower stabilization phase (185–187). However, the physicochemical evolution of respiratory droplets during evaporation is more complex than a wet-dry transition. As water is lost, droplets can pass through highly concentrated liquid states and may undergo liquid-liquid phase separation, changes in viscosity, or salt crystallization depending on relative humidity and solute composition (188, 189). These evolving phase states influence the chemical microenvironment experienced by viruses and can contribute to the heterogeneous decay patterns observed in IRPs. In the initial state right after particulate generation, evaporation rapidly concentrates non-volatile solutes and can alter droplet pH and phase state, creating a chemically dynamic environment in which viruses often undergo an early loss of infectivity. Current evidence supports roles for rapid physicochemical changes such as pH shifts, salt crystallization, and phase transitions; for IAV, acidic conditions relevant to exhaled particles have been shown to cause early infectivity loss through premature conformational changes in hemagglutinin (190, 191). For SARS-CoV-2, early airborne inactivation has been linked to droplet microphysics and pH changes (190, 192–195).

Biological components within droplets can counteract this instability. Mucins, and proteins form protective microenvironments that partially shield viruses from the osmotic and thermal shocks. This stabilizing effect has been observed in IAV, where mucins colocalize with virions and preserve infectivity, and in SARS-CoV-2, where elevated mucin concentrations enhance viral stability in IRPs (196, 197). Similarly, airborne phage and IAV studies showed that protein supplementation, such as adding bovine serum albumin, improved viral recovery (23, 198).

As evaporation progresses, droplets enter a semi-solid or dry phase, in which viral decay slows dramatically. Multiple studies confirm this wet-to-dry transition in the decay kinetics of enveloped viruses, with faster inactivation during the wet phase and substantially slower decay once droplets have desiccated (185, 194, 199, 200). Smaller droplets enter the dry phase more rapidly because of their high surface-area-to-volume ratio, enabling particles to carry viable virions for longer periods.

Immediately after emission, respiratory particles undergo a rapid physicochemical transition that determines how long virions remain infectious outside the host. Viruses that better tolerate this transition retain infectivity within suspended particles for longer, extending opportunities for inhalation exposure, particularly in poorly ventilated indoor environments (177, 201). Environmental stability should therefore be viewed as a temporal filter between emission and exposure: rather than determining whether transmission occurs, it defines the window during which emitted virions remain capable of initiating infection in the next host. This is consistent with the large indoor outbreaks described for viruses such as SARS-CoV-2 and measles virus (129, 132, 177, 202, 203). However, it is important to note that such clustering is not solely determined by downstream aerosol processes, but also reflects variability in viral emission between individuals. In this context, airborne stability should be considered as a factor that enables transmission once particles are emitted, rather than as the primary driver of superspreading events.

### Environmental stability

Once suspended in air, viral persistence depends on temperature, humidity, solute dynamics, and droplet composition (Fig. 2). Higher ambient temperatures accelerate viral decay by denaturing proteins, destabilizing lipid membranes in enveloped viruses, and accelerating RNA degradation. Ambient temperatures above ~30°C–40°C can inactivate enveloped viruses rapidly (204–206).

Historical aerosol studies have grouped virus survival responses to RH into several patterns, including improved survival at high RH, improved survival at low RH, a minimum at intermediate RH, or little apparent RH dependence. However, these patterns are not universal and depend strongly on the virus studied, the RH range tested, the exposure time, and the composition of the particles. For example, classic experiments with influenza A/WSNH aerosolized from cell-culture medium or allantoic fluid reported the lowest survival at intermediate RH (207), whereas more recent work with A(H1N1)pdm09 suspended in extracellular material from primary human bronchial epithelial cultures found little loss of infectivity across 23%–98% RH (198). SARS-CoV-2 likewise shows medium-dependent behavior rather than a single canonical RH profile: different decay rates have been reported in tissue-culture medium versus artificial saliva, and some studies found no independent effect of RH over approximately 20%–70% RH (194, 208, 209). By contrast, the limited experimental literature on MeV indicates poorer airborne survival at higher RH (210), while classic studies of foot-and-mouth disease virus found greater stability above roughly 50%–55% RH in some media (211).

Accordingly, the frequently cited U-shaped RH-viability profile is best regarded as a classical pattern observed in some influenza virus studies performed in relatively simple media, rather than a general rule for all enveloped respiratory viruses. Current evidence suggests that RH influences viral viability through coupled effects on evaporation, solute concentration, phase behavior, pH, and the protective or deleterious properties of the surrounding medium. At intermediate RH, droplets can remain concentrated, whereas at low RH efflorescence may occur; however, whether either state is more damaging depends on the virus and suspension medium (194, 195, 198, 212, 213).

For a summary of this section, see Box 3.

Box 3. **In the air**

Droplet transformation after exhalation

- Droplet origin determines droplet size; size governs airborne persistence, travel distance, and viable viral load.

Environmental stability

- Environmental conditions reshape infectivity: temperature, humidity, and solute concentration dictate how long expelled virions remain viable.

## CRITICAL KNOWLEDGE GAPS

Despite major advances in respiratory virology, many fundamental aspects of airborne transmission remain unresolved. The following eight gaps summarize where conceptual and technical limitations continue to constrain the field and where progress will depend on integrating virology, physics, and host biology. Now, in the period between pandemics, we have both the tools and the perspective needed to build a comprehensive, virus-agnostic understanding of the processes that enable a virion to achieve airborne transmission and, therefore, increased pandemic potential.

### Cell to air transition

Despite major advances in respiratory virology, the mechanistic steps that allow newly formed virions to leave infected cells and enter the airway lumen remain largely unresolved. While virus entry into epithelial cells is increasingly well characterized, the reverse path, from cellular exit to incorporation into exhaled particles, is still poorly understood. We lack a unified view of how virions travel through the periciliary layer, interact with the mucus gel, and encounter the mechanical forces that ultimately determine whether they are expelled into the air. Most phases of transmission have been studied in isolation, but the transitions connecting them are missing. In addition, mechanistic transmission studies remain heavily focused on a limited set of viruses, particularly influenza viruses and coronaviruses, highlighting the need to extend these approaches to a broader range of respiratory pathogens. Addressing this gap requires interdisciplinary approaches that combine molecular biophysics, advanced imaging of live airway epithelia, and aerosol science. Emerging technologies, such as real-time single-particle tracking and high-resolution visualization of mucus dynamics, are beginning to make these steps experimentally accessible (41, 214, 215). For example, single-particle tracking studies using fluorescent nanoparticles in human airway mucus have resolved how pore size, crosslinking, and charge govern particle mobility within native mucus gels, provide quantitative frameworks for studying virus-sized particle mobility in airway mucus (216).

### Species differentiation and zoonotic barriers

Cross-species transmission remains unpredictable. Most studies focus on receptor compatibility, yet mucus composition, glycan repertoires, airway architecture, and innate immune landscapes differ between species (51, 217, 218), and may shape how efficiently viruses are released into the air, transported along the tract, and stabilized. Current animal models capture only part of this complexity. Ferrets, hamsters, and non-human primates are invaluable for dissecting pathogenesis and basic transmission (219, 220). However, the species difference is compounded when virus stocks are not grown in a biologically matched system: growth in eggs or non-physiological cell lines can select for adaptations in glycoproteins or receptor usage that optimize replication in the production host, but distort transmission phenotypes in the target species (as shown, for example, in RSV and IAV work [221, 222]). Comparative studies that systematically map mucosal biochemistry, glycans, and innate responses across species, ideally using viruses grown in host-matched systems, are therefore essential. They would not only clarify why some viruses cross species barriers and acquire new transmission modes, but also help define when animal transmission data can be reliably extrapolated to humans and when it cannot.

### Influence of host variability

Age, sex, hormonal status, and genetic background modulate airway surface properties, receptor expression, and mucociliary function (223–225). However, host variability also shapes transmission through demographic structure and contact behavior, factors that remain poorly integrated into mechanistic studies of respiratory virus spread. Across respiratory viruses, age consistently emerges as one of the strongest determinants of transmission dynamics. School-aged children often act as major amplifiers of epidemics because of highly assortative contact patterns and dense mixing in educational settings, and they frequently introduce infections into households where transmission to other age groups occurs (183, 226–229). This pattern has been documented across multiple viruses, including influenza, RSV, MeV, and SARS-CoV-2, although the relative contributions of susceptibility, infectiousness, and contact structure vary between pathogens.

For example, influenza transmission studies consistently show that children have higher susceptibility and frequently seed household outbreaks (227, 230), while RSV infections in infants are often introduced by older siblings attending school (229, 231). Similarly, modern analyses of measles outbreaks in post-elimination settings continue to identify school-aged individuals as key transmission groups when immunity gaps arise (226). In contrast, early household studies of SARS-CoV-2 suggested that children were less susceptible than adults (232–234), illustrating that age effects can differ across pathogens and epidemiological contexts. By comparison, the influence of biological sex on transmission remains much less clear (234). While sex differences in immune responses and disease severity are well documented for several respiratory viruses (235), consistent evidence that males or females differ intrinsically in their transmissibility is limited. Observed differences in transmission are often small and difficult to disentangle from behavioral factors such as caregiving roles, occupational exposure, or household structure. Despite these insights, most experimental models of respiratory virus transmission rely on standardized cell lines, homogeneous airway cultures, or inbred animal models that overlook host heterogeneity. Addressing this gap will require experimental systems that incorporate human variability, including airway organoids and air-liquid interface cultures derived from donors of different ages, sexes, and genetic backgrounds, combined with epidemiological and contact-network data. Such approaches would enable direct testing of how host biological diversity and social structure together influence viral shedding, susceptibility, and transmission.

### Infectious dose

Although the theoretical concept of infectious dose is well established, quantitative dose-response relationships remain poorly defined for most respiratory viruses under natural exposure conditions. Experimental determination is challenging because controlled human dose-response studies are ethically constrained, while animal models only partially recapitulate human respiratory physiology and transmission dynamics. Furthermore, infectious dose is not a fixed property of a virus, but is influenced by factors such as the route of exposure, particle size, deposition site, host susceptibility, and the local airway environment. An alternative approach is the development of quantitative models that integrate both physical and biological determinants of transmission, including airflow, respiratory particle behavior, mucus properties, receptor accessibility, and host responses, to estimate the probability of infection (28, 29, 119, 136, 139). Such models already provide a valuable framework for understanding transmission risk, but their predictive accuracy will depend on continued improvements in our mechanistic understanding of viral entry, replication, release, and environmental persistence. Bridging these disciplines will be essential for developing more realistic dose-response models and improving predictions of respiratory virus transmission.

### Origin of the transmitted virus within the host

It remains unclear which cells and anatomical regions contribute most to the infectious material expelled during breathing, speaking, or coughing. Some viruses replicate efficiently but are rarely detected in aerosols, suggesting that replication alone is not sufficient for transmission shedding (159, 236, 237). Identifying the sources of exhaled virions requires spatially resolved virological and biophysical analyses, including: sampling approaches that differentiate URT vs LRT contributions; single-cell and region-specific viral load mapping. For example, bioluminescent and fluorescent reporter influenza viruses have enabled real-time spatial tracking of viral replication *in vivo*, whereas genetically barcoded influenza viruses have been used to trace transmission bottlenecks and identify the viral populations successfully transmitted between hosts (159, 238–240). Although the necessary technologies now exist, these experiments remain technically demanding: obtaining reproducible data that accurately link cellular origin to airborne output, and translating these findings from animal models to human respiratory virus transmission, remain major experimental challenges.

### Virus-host interactions determining transmission failures

One of the most persistent open questions in respiratory virology is why some viruses can replicate to high titers in the respiratory tract yet show little or no efficient onward transmission. This disconnect between replication and spread has been observed in several virus-host contexts, including pathogens that can be detected in respiratory tissues or secretions but do not transmit readily through the air, such as Ebola virus or mpox virus (241–243). These observations indicate that the presence of virus in the respiratory tract is not, by itself, sufficient to confer transmissibility. This discrepancy likely reflects differences in egress polarity, glycan binding, and envelope composition, which determine whether virions access the mucus layer and become aerosolized. Another plausible explanation for the lack of airborne transmission of mpox virus, for instance, could be the extended exposure duration required (typically exceeding 24 hours of continuous exposure) to deliver a viral load equivalent to the infectious dose into the airway of an exposed individual, as recently suggested by a CFD-based study (29). In general, addressing the transmissibility question remains technically challenging, as it requires disentangling which viral and host factors truly govern the transition from replication to release. We need to identify those that ultimately enable airborne transmission and pandemic potential. Understanding why some viruses that replicate in the same anatomical region fail to transmit efficiently is therefore central to predicting spillover risk, viral evolution, and the emergence of future pandemics.

### Virus-host interactions determining stability

Environmental stability is increasingly understood as an emergent property of the virion and the surrounding particle microenvironment, including airway surface liquid, saliva, mucins, and even commensal microorganisms (193). At the same time, key mechanistic questions remain unresolved, particularly how particle microphysics, chemical changes, and virus-milieu interactions combine to damage specific viral components under physiologically relevant conditions. Addressing these gaps will require interdisciplinary approaches that couple aerosol physics with virological readouts in realistic human-derived matrices. Priority areas include integrated chamber systems that simultaneously measure particle dynamics, pH, phase state, and infectivity; experiments using donor-derived respiratory fluids and airway epithelial secretions; structural and molecular assays that identify the lesions responsible for loss of infectivity; and human-relevant ALI, organoid, or lung-on-chip models exposed to aerosols rather than liquid inoculum. Linking these controlled studies with indoor air surveillance and exposure modeling will be essential for translating laboratory stability measurements into a predictive framework for real-world transmission (194, 195, 198, 213, 244, 245).

### Viral preference for specific droplet types

Droplets generated during exhalation are not homogeneous: their size, solute content, and mucus composition vary widely (171, 246, 247). Whether certain viruses preferentially associate with specific droplet fractions, those richer in mucins, salts, or surfactant, remains an open question. Addressing this question will require quantitative assays that measure viral load across size-resolved droplets, biochemical profiling of droplet subfractions, and experimental systems that manipulate mucus, salt, and surfactant composition to test how these parameters shape viral partitioning. For example, cascade impactors have been used to collect human exhaled particles in discrete size fractions and quantify viral RNA and infectious virus for influenza and SARS-CoV-2, revealing that viral material is often enriched in particles < 5 µm without resolving droplet-specific biochemical drivers (178, 248). These approaches will clarify whether specific droplet types disproportionately contribute to transmission.

### Conclusion

Addressing these gaps will require both conceptual integration and technical innovation. Mechanistic insight must evolve from descriptive to predictive, combining high-resolution imaging, quantitative modeling, and controlled human data. The current inter-respiratory virus pandemic period offers a unique opportunity to establish cross-disciplinary collaborations that unify molecular biology, host physiology, and aerobiology. Understanding early transmission of respiratory viruses is critical for anticipating and preventing future ones. The COVID-19 pandemic made this painfully clear: early misjudgments about airborne transmission delayed essential interventions like indoor ventilation and high-filtration masks, contributing to widespread global transmission. Where and how a virus enters the body, the infectious dose required, and the host’s immune response all shape its ability to spread. Yet without a mechanistic understanding of these processes, epidemiological data alone leave us one step behind. If we fail to grasp what enables a virus to go airborne, we risk underestimating threats and repeating the same delays in response. Bridging the gaps between virology, immunology, and aerosol science is essential to refine predictive models, inform timely public health measures, and build resilience against emerging respiratory pathogens.

## References

[1] Li Y, Wang X, Blau DM, Caballero MT, Feikin DR, Gill CJ, Madhi SA, Omer SB, Simões EAF, Campbell H, et al.. 2022. Global, regional, and national disease burden estimates of acute lower respiratory infections due to respiratory syncytial virus in children younger than 5 years in 2019: a systematic analysis. The Lancet 399:2047–2064. doi:10.1016/S0140-6736(22)00478-0 PMC7613574 35598608

[2] Iuliano AD, Roguski KM, Chang HH, Muscatello DJ, Palekar R, Tempia S, Cohen C, Gran JM, Schanzer D, Cowling BJ, et al.. 2018. Estimates of global seasonal influenza-associated respiratory mortality: a modelling study. Lancet 391:1285–1300. doi:10.1016/S0140-6736(17)33293-2 PMC5935243 29248255

[3] World Health Organization. 2024. COVID-19 epidemiological update: special edition. World Health Organization.

[4] World Health Organization. 2026. WHO coronavirus (COVID-19) dashboard: deaths. Available from: https://data.who.int/dashboards/covid19/deaths

[5] Kyu HH, Vongpradith A, Sirota SB, Novotney A, Troeger CE, Doxey MC, Bender RG, Ledesma JR, Biehl MH, Albertson SB, et al.. 2022. Age–sex differences in the global burden of lower respiratory infections and risk factors, 1990–2019: results from the Global Burden of Disease Study 2019. Lancet Infect Dis 22:1626–1647. doi:10.1016/S1473-3099(22)00510-2 PMC9605880 35964613

[6] Tejada-Vera B, Bastian B, Curtin S. 2025. Deaths: leading causes for 2023. National Vital Statistics Reports 74(10) 10.15620/cdc/174607 PMC12498176 41021892

[7] Molinari N-AM, Ortega-Sanchez IR, Messonnier ML, Thompson WW, Wortley PM, Weintraub E, Bridges CB. 2007. The annual impact of seasonal influenza in the US: measuring disease burden and costs. Vaccine 25:5086–5096. doi:10.1016/j.vaccine.2007.03.046 17544181

[8] Bourouiba L. 2020. Turbulent gas clouds and respiratory pathogen emissions. JAMA. doi:10.1001/jama.2020.4756 32215590

[9] Faramarzi A, Norouzi S, Dehdarirad H, Aghlmand S, Yusefzadeh H, Javan-Noughabi J. 2024. The global economic burden of COVID-19 disease: a comprehensive systematic review and meta-analysis. Syst Rev 13:68. doi:10.1186/s13643-024-02476-6 PMC10870589 38365735

[10] Putri WCWS, Muscatello DJ, Stockwell MS, Newall AT. 2018. Economic burden of seasonal influenza in the United States. Vaccine 36:3960–3966. doi:10.1016/j.vaccine.2018.05.057 29801998

[11] World Health Organization. 2024. Global technical consultation report on proposed terminology for pathogens that transmit through the air. Geneva, Switzerland World Health Organization

[12] Duguid JP. 1946. The size and the duration of air-carriage of respiratory droplets and droplet-nuclei. Epidemiol Infect 44:471–479. doi:10.1017/S0022172400019288 PMC2234804 20475760

[13] Johnson GR, Morawska L. 2009. The mechanism of breath aerosol formation. J Aerosol Med Pulm Drug Deliv 22:229–237. doi:10.1089/jamp.2008.0720 19415984

[14] Hinds WC. 1999. Aerosol technology: properties, behavior, and measurement of airborne particles. 2nd edition ed, p 452. John Wiley & Sons, New York, NY, USA.

[15] Milton DK. 2020. A rosetta stone for understanding infectious drops and aerosols. J Pediatric Infect Dis Soc 9:413–415. doi:10.1093/jpids/piaa079 PMC7495905 32706376

[16] Nogrady B. 2024. WHO redefines airborne transmission: what does that mean for future pandemics? Nature. doi:10.1038/d41586-024-01173-7 38658721

[17] Jimenez JL, Marr LC, Randall K, Ewing ET, Tufekci Z, Greenhalgh T, Tellier R, Tang JW, Li Y, Morawska L, Mesiano-Crookston J, Fisman D, Hegarty O, Dancer SJ, Bluyssen PM, Buonanno G, Loomans MGLC, Bahnfleth WP, Yao M, Sekhar C, Wargocki P, Melikov AK, Prather KA. 2022. What were the historical reasons for the resistance to recognizing airborne transmission during the COVID-19 pandemic? Indoor Air 32:e13070. doi:10.1111/ina.13070 PMC9538841 36040283

[18] Zimmer C. 2025. Air-borne: the hidden history of the life we breathe. Dutton, New York.

[19] Wells WF. 1934. On air-borne infection. Am J Epidemiol 20:611–618. doi:10.1093/oxfordjournals.aje.a118097

[20] Heyder J, Gebhart J, Rudolf G, Schiller CF, Stahlhofen W. 1986. Deposition of particles in the human respiratory tract in the size range 0.005–15 μm. J Aerosol Sci 17:811–825. doi:10.1016/0021-8502(86)90035-2

[21] Raabe OG. 1988. Regional deposition of inhaled monodisperse coarse and fine aerosol particles in small laboratory animals. Ann Occup Hyg:53–63. doi:10.1093/annhyg/32.inhaled_particles_VI.53

[22] Basu S. 2025. On the mechanics of inhaled bronchial transmission of pathogenic microdroplets generated from the upper respiratory tract, with implications for downwind infection onset. PLoS ONE 20:e0335962. doi:10.1371/journal.pone.0335962 PMC12594396 41202071

[23] Humphrey B, Tezak M, Lobitz M, Hendricks A, Sanchez A, Zenker J, Storch S, Davis RD, Ricken B, Cahill J. 2023. Viral preservation with protein-supplemented nebulizing media in aerosols. Appl Environ Microbiol 89:e01545–22. doi:10.1128/aem.01545-22 PMC10057872 36856430

[24] Basu S, Akash MMH, Hochberg NS, Senior BA, Joseph-McCarthy D, Chakravarty A. 2022. From SARS-CoV-2 infection to COVID-19 morbidity: an *in silico* projection of virion flow rates to the lower airway via nasopharyngeal fluid boluses. RHINOL 5:10–18. doi:10.4193/RHINOL/21.053

[25] Chakravarty A, Panchagnula MV, Patankar NA. 2023. Inhalation of virus-loaded droplets as a clinically plausible pathway to deep lung infection. Front Physiol 14:14. doi:10.3389/fphys.2023.1073165 PMC9892651 36744036

[26] Haddrell AE, Davies JF, Reid JP. 2015. Dynamics of particle size on inhalation of environmental aerosol and impact on deposition fraction. Environ Sci Technol 49:14512–14521. doi:10.1021/acs.est.5b01930 26568475

[27] Scheuch G, Stahlhofen W. 1987. Particle deposition of inhaled aerosol boluses in the upper human airways. J Aerosol Sci 18:725–727. doi:10.1016/0021-8502(87)90107-8

[28] Basu S. 2021. Computational characterization of inhaled droplet transport to the nasopharynx. Sci Rep 11:6652. doi:10.1038/s41598-021-85765-7 PMC7988116 33758241

[29] Yeasin M, Akash MMH, Malakar A, Borojeni AAT, Tummala A, Wu J, Bennett WD, Bodnar WM, Kimbell JS, Chakravarty A, Port JR, Basu S. 2025. Decoding the mechanophysiology for inhaled onset of smallpox with model-based implications for mpox spread. Sci Rep 16:2142. doi:10.1038/s41598-025-31923-0 PMC12808741 41392049

[30] Hill DB, Button B, Rubinstein M, Boucher RC. 2022. Physiology and pathophysiology of human airway mucus. Physiol Rev 102:1757–1836. doi:10.1152/physrev.00004.2021 PMC9665957 35001665

[31] Button B, Cai L-H, Ehre C, Kesimer M, Hill DB, Sheehan JK, Boucher RC, Rubinstein M. 2012. A periciliary brush promotes the lung health by separating the mucus layer from airway epithelia. Science 337:937–941. doi:10.1126/science.1223012 PMC3633213 22923574

[32] Okuda K, Chen G, Subramani DB, Wolf M, Gilmore RC, Kato T, Radicioni G, Kesimer M, Chua M, Dang H, Livraghi-Butrico A, Ehre C, Doerschuk CM, Randell SH, Matsui H, Nagase T, O’Neal WK, Boucher RC. 2019. Localization of secretory mucins MUC5AC and MUC5B in normal/healthy human airways. Am J Respir Crit Care Med 199:715–727. doi:10.1164/rccm.201804-0734OC PMC6423099 30352166

[33] Zajac M, Dreano E, Edwards A, Planelles G, Sermet-Gaudelus I. 2021. Airway surface liquid pH regulation in airway epithelium current understandings and gaps in knowledge. Int J Mol Sci 22:3384. doi:10.3390/ijms22073384 PMC8037888 33806154

[34] Knowles MR, Robinson JM, Wood RE, Pue CA, Mentz WM, Wager GC, Gatzy JT, Boucher RC. 1997. Ion composition of airway surface liquid of patients with cystic fibrosis as compared with normal and disease-control subjects. J Clin Invest 100:2588–2595. doi:10.1172/JCI119802 PMC508460 9366574

[35] Song Y, Thiagarajah J, Verkman AS. 2003. Sodium and chloride concentrations, pH, and depth of airway surface liquid in distal airways. J Gen Physiol 122:511–519. doi:10.1085/jgp.200308866 PMC2229580 14557401

[36] Fahy JV, Dickey BF. 2010. Airway mucus function and dysfunction. N Engl J Med 363:2233–2247. doi:10.1056/NEJMra0910061 PMC4048736 21121836

[37] Hill DB, Long RF, Kissner WJ, Atieh E, Garbarine IC, Markovetz MR, Fontana NC, Christy M, Habibpour M, Tarran R, Forest MG, Boucher RC, Button B. 2018. Pathological mucus and impaired mucus clearance in cystic fibrosis patients result from increased concentration, not altered pH. Eur Respir J 52:1801297. doi:10.1183/13993003.01297-2018 PMC6446239 30361244

[38] Porfiryeva NN, Zlotver I, Sosnik A. 2025. Combined effect of size and charge on the interaction of nanoparticles with mucus-mimicking mucin hydrogels. Pharmaceuticals (Basel) 18:1498. doi:10.3390/ph18101498 PMC12567317 41155613

[39] Cohen M, Zhang X-Q, Senaati HP, Chen H-W, Varki NM, Schooley RT, Gagneux P. 2013. Influenza A penetrates host mucus by cleaving sialic acids with neuraminidase. Virol J 10:321. doi:10.1186/1743-422X-10-321 PMC3842836 24261589

[40] Iseli AN, Pohl MO, Glas I, Gaggioli E, Martínez-Barragán P, David SC, Schaub A, Luo B, Klein LK, Bluvshtein N, Violaki K, Motos G, Hugentobler W, Nenes A, Krieger UK, Peter T, Kohn T, Stertz S. 2023. The neuraminidase activity of influenza A virus determines the strain-specific sensitivity to neutralization by respiratory mucus. J Virol 97:e0127123. doi:10.1128/jvi.01271-23 PMC10617592 37819131

[41] Kaler L, Engle EM, Corkran M, Iverson E, Boboltz A, Ignacio MA, Yeruva T, Scull MA, Duncan GA. 2025. Mucus physically restricts influenza A viral particle access to the epithelium. Adv Biol (Weinh) 9:e2400329. doi:10.1002/adbi.202400329 PMC12001005 39936480

[42] Kaler L, Iverson E, Bader S, Song D, Scull MA, Duncan GA. 2022. Influenza A virus diffusion through mucus gel networks. Commun Biol 5. doi:10.1038/s42003-022-03204-3 PMC8941132 35318436

[43] Lang Y, Li W, Li Z, Koerhuis D, van den Burg ACS, Rozemuller E, Bosch B-J, van Kuppeveld FJM, Boons G-J, Huizinga EG, van der Schaar HM, de Groot RJ. 2020. Coronavirus hemagglutinin-esterase and spike proteins coevolve for functional balance and optimal virion avidity. Proc Natl Acad Sci USA 117:25759–25770. doi:10.1073/pnas.2006299117 PMC7568303 32994342

[44] Desforges M, Desjardins J, Zhang C, Talbot PJ. 2013. The acetyl-esterase activity of the hemagglutinin-esterase protein of human coronavirus OC43 strongly enhances the production of infectious virus. J Virol 87:3097–3107. doi:10.1128/JVI.02699-12 PMC3592170 23283955

[45] Huang X, Dong W, Milewska A, Golda A, Qi Y, Zhu QK, Marasco WA, Baric RS, Sims AC, Pyrc K, Li W, Sui J. 2015. Human coronavirus HKU1 spike protein uses O-acetylated sialic acid as an attachment receptor determinant and employs hemagglutinin-esterase protein as a receptor-destroying enzyme. J Virol 89:7202–7213. doi:10.1128/JVI.00854-15 PMC4473545 25926653

[46] Morein B, Bergman R. 1972. Effect of parainfluenza-3 neuraminidase on bovine nasal secretion. Infect Immun 6:174–177. doi:10.1128/iai.6.2.174-177.1972 PMC422511 4347547

[47] Höglund S, Morein B. 1974. Effect of parainfluenza-3 virus neuraminidase on the structure of the gel phase of bovine nasal secretion. Infect Immun 10:877–882. doi:10.1128/iai.10.4.877-882.1974 PMC423035 4372181

[48] Yang X, Forier K, Steukers L, Van Vlierberghe S, Dubruel P, Braeckmans K, Glorieux S, Nauwynck HJ. 2012. Immobilization of pseudorabies virus in porcine tracheal respiratory mucus revealed by single particle tracking. PLoS One 7:e51054. doi:10.1371/journal.pone.0051054 PMC3517622 23236432

[49] Wardzala CL, Wood AM, Belnap DM, Kramer JR. 2022. Mucins inhibit coronavirus infection in a glycan-dependent manner. ACS Cent Sci 8:351–360. doi:10.1021/acscentsci.1c01369 PMC8864775 35345395

[50] Cortjens B, de Boer OJ, de Jong R, Antonis AF, Sabogal Piñeros YS, Lutter R, van Woensel JB, Bem RA. 2016. Neutrophil extracellular traps cause airway obstruction during respiratory syncytial virus disease. J Pathol 238:401–411. doi:10.1002/path.4660 26468056

[51] Walther T, Karamanska R, Chan RWY, Chan MCW, Jia N, Air G, Hopton C, Wong MP, Dell A, Malik Peiris JS, Haslam SM, Nicholls JM. 2013. Glycomic analysis of human respiratory tract tissues and correlation with influenza virus infection. PLoS Pathog 9:e1003223. doi:10.1371/journal.ppat.1003223 PMC3597497 23516363

[52] Jia N, Barclay WS, Roberts K, Yen H-L, Chan RWY, Lam AKY, Air G, Peiris JSM, Dell A, Nicholls JM, Haslam SM. 2014. Glycomic characterization of respiratory tract tissues of ferrets. J Biol Chem 289:28489–28504. doi:10.1074/jbc.M114.588541 PMC4192499 25135641

[53] Jayaraman A, Chandrasekaran A, Viswanathan K, Raman R, Fox JG, Sasisekharan R. 2012. Decoding the distribution of glycan receptors for human-adapted influenza A viruses in ferret respiratory tract. PLoS One 7:e27517. doi:10.1371/journal.pone.0027517 PMC3281014 22359533

[54] Linssen RS, Chai G, Ma J, Kummarapurugu AB, van Woensel JBM, Bem RA, Kaler L, Duncan GA, Zhou L, Rubin BK, Xu Q. 2021. Neutrophil extracellular traps increase airway mucus viscoelasticity and slow mucus particle transit. Am J Respir Cell Mol Biol 64:69–78. doi:10.1165/rcmb.2020-0168OC PMC7780998 33095650

[55] Abrami M, Biasin A, Tescione F, Tierno D, Dapas B, Carbone A, Grassi G, Conese M, Di Gioia S, Larobina D, Grassi M. 2024. Mucus structure, viscoelastic properties, and composition in chronic respiratory diseases. Int J Mol Sci 25:1933. doi:10.3390/ijms25031933 PMC10856136 38339210

[56] Rulff H, Schmidt RF, Wei L-F, Fentker K, Kerkhoff Y, Mertins P, Mall MA, Lauster D, Gradzielski M. 2024. Comprehensive characterization of the viscoelastic properties of bovine submaxillary mucin (BSM) hydrogels and the effect of additives. Biomacromolecules 25:4014–4029. doi:10.1021/acs.biomac.4c00153 PMC11238336 38832927

[57] Becker ME, Martin-Sancho L, Simons LM, McRaven MD, Chanda SK, Hultquist JF, Hope TJ. 2024. Live imaging of airway epithelium reveals that mucociliary clearance modulates SARS-CoV-2 spread. Nat Commun 15:9480. doi:10.1038/s41467-024-53791-4 PMC11531594 39488529

[58] Robinot R, Hubert M, de Melo GD, Lazarini F, Bruel T, Smith N, Levallois S, Larrous F, Fernandes J, Gellenoncourt S, Rigaud S, Gorgette O, Thouvenot C, Trébeau C, Mallet A, Duménil G, Gobaa S, Etournay R, Lledo P-M, Lecuit M, Bourhy H, Duffy D, Michel V, Schwartz O, Chakrabarti LA. 2021. SARS-CoV-2 infection induces the dedifferentiation of multiciliated cells and impairs mucociliary clearance. Nat Commun 12:4354. doi:10.1038/s41467-021-24521-x PMC8285531 34272374

[59] Smith CM, Kulkarni H, Radhakrishnan P, Rutman A, Bankart MJ, Williams G, Hirst RA, Easton AJ, Andrew PW, O’Callaghan C. 2014. Ciliary dyskinesia is an early feature of respiratory syncytial virus infection. Eur Respir J 43:485–496. doi:10.1183/09031936.00205312 23520320

[60] Kamiya Y, Fujisawa T, Katsumata M, Yasui H, Suzuki Y, Karayama M, Hozumi H, Furuhashi K, Enomoto N, Nakamura Y, Inui N, Setou M, Ito M, Suzuki T, Ikegami K, Suda T. 2020. Influenza A virus enhances ciliary activity and mucociliary clearance via TLR3 in airway epithelium. Respir Res 21:282. doi:10.1186/s12931-020-01555-1 PMC7590254 33109186

[61] Sakai T, Nishimura SI, Naito T, Saito M. 2017. Influenza A virus hemagglutinin and neuraminidase act as novel motile machinery. Sci Rep 7:45043. doi:10.1038/srep45043 PMC5366856 28344335

[62] Liu M, de Vries E, de Haan CAM. 2025. Virion motility of sialoglycan-cleaving respiratory viruses. Npj Viruses 3:59. doi:10.1038/s44298-025-00140-x PMC12304231 40721499

[63] Guo H, Rabouw H, Slomp A, Dai M, van der Vegt F, van Lent JWM, McBride R, Paulson JC, de Groot RJ, van Kuppeveld FJM, de Vries E, de Haan CAM. 2018. Kinetic analysis of the influenza A virus HA/NA balance reveals contribution of NA to virus-receptor binding and NA-dependent rolling on receptor-containing surfaces. PLOS Pathog 14:e1007233. doi:10.1371/journal.ppat.1007233 PMC6107293 30102740

[64] Clary-Meinesz CF, Cosson J, Huitorel P, Blaive B. 1992. Temperature effect on the ciliary beat frequency of human nasal and tracheal ciliated cells. Biol Cell 76:335–338. doi:10.1016/0248-4900(92)90436-5 1305479

[65] Schmid A, Sutto Z, Nlend M-C, Horvath G, Schmid N, Buck J, Levin LR, Conner GE, Fregien N, Salathe M. 2007. Soluble adenylyl cyclase is localized to cilia and contributes to ciliary beat frequency regulation via production of cAMP. J Gen Physiol 130:99–109. doi:10.1085/jgp.200709784 PMC2154360 17591988

[66] Simet SM, Sisson JH, Pavlik JA, Devasure JM, Boyer C, Liu X, Kawasaki S, Sharp JG, Rennard SI, Wyatt TA. 2010. Long-term cigarette smoke exposure in a mouse model of ciliated epithelial cell function. Am J Respir Cell Mol Biol 43:635–640. doi:10.1165/rcmb.2009-0297OC PMC2993085 20042711

[67] Milara J, Armengot M, Bañuls P, Tenor H, Beume R, Artigues E, Cortijo J. 2012. Roflumilast N-oxide, a PDE4 inhibitor, improves cilia motility and ciliated human bronchial epithelial cells compromised by cigarette smoke *in vitro*. Br J Pharmacol 166:2243–2262. doi:10.1111/j.1476-5381.2012.01929.x PMC3448891 22385203

[68] Brierley L, Pedersen AB, Woolhouse MEJ. 2019. Tissue tropism and transmission ecology predict virulence of human RNA viruses. PLOS Biol 17:e3000206. doi:10.1371/journal.pbio.3000206 PMC6879112 31770368

[69] Zeng H, Goldsmith CS, Maines TR, Belser JA, Gustin KM, Pekosz A, Zaki SR, Katz JM, Tumpey TM. 2013. Tropism and infectivity of influenza virus, including highly pathogenic avian H5N1 virus, in ferret tracheal differentiated primary epithelial cell cultures. J Virol 87:2597–2607. doi:10.1128/JVI.02885-12 PMC3571379 23255802

[70] Morgan AL, Vu MN, Zhou Y, Lokugamage KG, Meyers WM, Alvarado RE, Ahearn Y, Estes LK, Plante JA, Johnson BA, Suthar MS, Walker DH, Plante KS, Menachery VD. 2025. The furin cleavage site is required for pathogenesis, but not transmission, of SARS-CoV-2. J Virol 99:e0046725. doi:10.1128/jvi.00467-25 PMC12282137 40492735

[71] van Riel D, den Bakker MA, Leijten LME, Chutinimitkul S, Munster VJ, de Wit E, Rimmelzwaan GF, Fouchier RAM, Osterhaus ADME, Kuiken T. 2010. Seasonal and pandemic human influenza viruses attach better to human upper respiratory tract epithelium than avian influenza viruses. Am J Pathol 176:1614–1618. doi:10.2353/ajpath.2010.090949 PMC2843453 20167867

[72] Otter CJ, Fausto A, Tan LH, Khosla AS, Cohen NA, Weiss SR. 2023. Infection of primary nasal epithelial cells differentiates among lethal and seasonal human coronaviruses. Proc Natl Acad Sci USA 120:15. doi:10.1073/pnas.2218083120 PMC10104492 37023127

[73] Ahn JH, Kim J, Hong SP, Choi SY, Yang MJ, Ju YS, Kim YT, Kim HM, Rahman MT, Chung MK, Hong SD, Bae H, Lee C-S, Koh GY. 2021. Nasal ciliated cells are primary targets for SARS-CoV-2 replication in the early stage of COVID-19. J Clin Invest 131:13. doi:10.1172/JCI148517 PMC8245175 34003804

[74] Hamming I, Timens W, Bulthuis M, Lely A, Navis G, van Goor H. 2004. Tissue distribution of ACE2 protein, the functional receptor for SARS coronavirus. A first step in understanding SARS pathogenesis. J Pathol 203:631–637. doi:10.1002/path.1570 PMC7167720 15141377

[75] Sims AC, Baric RS, Yount B, Burkett SE, Collins PL, Pickles RJ. 2005. Severe acute respiratory syndrome coronavirus infection of human ciliated airway epithelia: role of ciliated cells in viral spread in the conducting airways of the lungs. J Virol 79:15511–15524. doi:10.1128/JVI.79.24.15511-15524.2005 PMC1316022 16306622

[76] Nicholls JM, Butany J, Poon LLM, Chan KH, Beh SL, Poutanen S, Peiris JSM, Wong M. 2006. Time course and cellular localization of SARS-CoV nucleoprotein and RNA in lungs from fatal cases of SARS. PLoS Med 3:e27. doi:10.1371/journal.pmed.0030027 PMC1324951 16379499

[77] Ludlow M, Lemon K, de Vries RD, McQuaid S, Millar EL, van Amerongen G, Yüksel S, Verburgh RJ, Osterhaus ADME, de Swart RL, Duprex WP. 2013. Measles virus infection of epithelial cells in the macaque upper respiratory tract is mediated by subepithelial immune cells. J Virol 87:4033–4042. doi:10.1128/JVI.03258-12 PMC3624209 23365435

[78] Ferreira CSA, Frenzke M, Leonard VHJ, Welstead GG, Richardson CD, Cattaneo R. 2010. Measles virus infection of alveolar macrophages and dendritic cells precedes spread to lymphatic organs in transgenic mice expressing human signaling lymphocytic activation molecule (SLAM, CD150). J Virol 84:3033–3042. doi:10.1128/JVI.01559-09 PMC2826031 20042501

[79] Singh BK, Li N, Mark AC, Mateo M, Cattaneo R, Sinn PL. 2016. Cell-to-cell contact and nectin-4 govern spread of measles virus from primary human myeloid cells to primary human airway epithelial cells. J Virol 90:6808–6817. doi:10.1128/JVI.00266-16 PMC4944272 27194761

[80] Laporte M, Raeymaekers V, Van Berwaer R, Vandeput J, Marchand-Casas I, Thibaut H-J, Van Looveren D, Martens K, Hoffmann M, Maes P, Pöhlmann S, Naesens L, Stevaert A. 2021. The SARS-CoV-2 and other human coronavirus spike proteins are fine-tuned towards temperature and proteases of the human airways. PLOS Pathog 17:e1009500. doi:10.1371/journal.ppat.1009500 PMC8061995 33886690

[81] Edwards DA, Man JC, Brand P, Katstra JP, Sommerer K, Stone HA, Nardell E, Scheuch G. 2004. Inhaling to mitigate exhaled bioaerosols. Proc Natl Acad Sci USA 101:17383–17388. doi:10.1073/pnas.0408159101 PMC536048 15583121

[82] Prévost J, Richard J, Gasser R, Ding S, Fage C, Anand SP, Adam D, Gupta Vergara N, Tauzin A, Benlarbi M, Gong SY, Goyette G, Privé A, Moreira S, Charest H, Roger M, Mothes W, Pazgier M, Brochiero E, Boivin G, Abrams CF, Schön A, Finzi A. 2021. Impact of temperature on the affinity of SARS-CoV-2 Spike glycoprotein for host ACE2. J Biol Chem 297:101151. doi:10.1016/j.jbc.2021.101151 PMC8406544 34478710

[83] Gong SY, Ding S, Benlarbi M, Chen Y, Vézina D, Marchitto L, Beaudoin-Bussières G, Goyette G, Bourassa C, Bo Y, Medjahed H, Levade I, Pazgier M, Côté M, Richard J, Prévost J, Finzi A. 2022. Temperature influences the interaction between SARS-CoV-2 spike from omicron subvariants and human ACE2. Viruses 14:2178. doi:10.3390/v14102178 PMC9607596 36298733

[84] V’kovski P, Gultom M, Kelly JN, Steiner S, Russeil J, Mangeat B, Cora E, Pezoldt J, Holwerda M, Kratzel A, Laloli L, Wider M, Portmann J, Tran T, Ebert N, Stalder H, Hartmann R, Gardeux V, Alpern D, Deplancke B, Thiel V, Dijkman R. 2021. Disparate temperature-dependent virus-host dynamics for SARS-CoV-2 and SARS-CoV in the human respiratory epithelium. PLoS Biol 19:e3001158. doi:10.1371/journal.pbio.3001158 PMC8032198 33780434

[85] Benlarbi M, Ding S, Bélanger É, Tauzin A, Poujol R, Medjahed H, El Ferri O, Bo Y, Bourassa C, Hussin J, Fafard J, Pazgier M, Levade I, Abrams C, Côté M, Finzi A. 2024. Temperature-dependent Spike-ACE2 interaction of omicron subvariants is associated with viral transmission. mBio 15. doi:10.1128/mbio.00907-24 PMC11323525 38953636

[86] Suzuki Y, Ito T, Suzuki T, Holland RE, Chambers TM, Kiso M, Ishida H, Kawaoka Y. 2000. Sialic acid species as a determinant of the host range of influenza A viruses. J Virol 74:11825–11831. doi:10.1128/jvi.74.24.11825-11831.2000 PMC112465 11090182

[87] Turnbull ML, Wang Y, Clare S, Lieber G, Williams SL, Noerenberg M, Alexander AJT, Clohisey Hendry S, Stewart DG, Hughes J, et al.. 2025. Avian-origin influenza A viruses tolerate elevated pyrexic temperatures in mammals. Science 390:eadq4691. doi:10.1126/science.adq4691 PMC7618609 41308154

[88] Bestle D, Heindl MR, Limburg H, Van Lam van T, Pilgram O, Moulton H, Stein DA, Hardes K, Eickmann M, Dolnik O, Rohde C, Klenk H-D, Garten W, Steinmetzer T, Böttcher-Friebertshäuser E. 2020. TMPRSS2 and furin are both essential for proteolytic activation of SARS-CoV-2 in human airway cells. Life Sci Alliance 3:e202000786. doi:10.26508/lsa.202000786 PMC7383062 32703818

[89] Koch J, Uckeley ZM, Doldan P, Stanifer M, Boulant S, Lozach P. 2021. TMPRSS2 expression dictates the entry route used by SARS‐CoV‐2 to infect host cells. EMBO J 40:16. doi:10.15252/embj.2021107821 PMC8365257 34159616

[90] Peacock TP, Goldhill DH, Zhou J, Baillon L, Frise R, Swann OC, Kugathasan R, Penn R, Brown JC, Sanchez-David RY, Braga L, Williamson MK, Hassard JA, Staller E, Hanley B, Osborn M, Giacca M, Davidson AD, Matthews DA, Barclay WS. 2021. The furin cleavage site in the SARS-CoV-2 spike protein is required for transmission in ferrets. Nat Microbiol 6:899–909. doi:10.1038/s41564-021-00908-w PMC7619196 33907312

[91] Meng B, Abdullahi A, Ferreira IATM, Goonawardane N, Saito A, Kimura I, Yamasoba D, Gerber PP, Fatihi S, Rathore S, et al.. 2022. Altered TMPRSS2 usage by SARS-CoV-2 omicron impacts infectivity and fusogenicity. Nature 603:706–714. doi:10.1038/s41586-022-04474-x PMC8942856 35104837

[92] Strobelt R, Broennimann K, Adler J, Shaul Y. 2023. SARS-CoV-2 omicron specific mutations affecting infectivity, fusogenicity, and partial TMPRSS2-independency. Viruses 15:1129. doi:10.3390/v15051129 PMC10223509 37243215

[93] Mykytyn AZ, Breugem TI, Geurts MH, Beumer J, Schipper D, van Acker R, van den Doel PB, van Royen ME, Zhang J, Clevers H, Haagmans BL, Lamers MM. 2023. SARS-CoV-2 omicron entry is type II transmembrane serine protease-mediated in human airway and intestinal organoid models. J Virol 97:e0085123. doi:10.1128/jvi.00851-23 PMC10506477 37555660

[94] Metzdorf K, Jacobsen H, Greweling-Pils MC, Hoffmann M, Lüddecke T, Miller F, Melcher L, Kempf AM, Nehlmeier I, Bruder D, Widera M, Ciesek S, Pöhlmann S, Čičin-Šain L. 2023. TMPRSS2 is essential for SARS-CoV-2 beta and omicron infection. Viruses 15:271. doi:10.3390/v15020271 PMC9961888 36851486

[95] Morris SE, Nguyen HQ, Grijalva CG, Hanson KE, Zhu Y, Biddle JE, Meece JK, Halasa NB, Chappell JD, Mellis AM, Reed C, Biggerstaff M, Belongia EA, Talbot HK, Rolfes MA. 2024. Influenza virus shedding and symptoms: dynamics and implications from a multiseason household transmission study. PNAS Nexus 3:pgae338. doi:10.1093/pnasnexus/pgae338 PMC11378077 39246667

[96] Otomaru H, Sornillo JBT, Kamigaki T, Bado SLP, Okamoto M, Saito-Obata M, Inobaya MT, Segubre-Mercado E, Alday PP, Saito M, Tallo VL, Quiambao BP, Oshitani H, Cook AR. 2021. Risk of transmission and viral shedding from the time of infection for respiratory syncytial virus in households. Am J Epidemiol 190:2536–2543. doi:10.1093/aje/kwab181 PMC8634588 34216204

[97] Shetty N, Shephard MJ, Rockey NC, Macenczak H, Traenkner J, Danzy S, Vargas-Maldonado N, Arts PJ, Le Sage V, Anderson EJ, Lyon GM, Fitts EC, Gulick DA, Mehta AK, El-Chami MF, Kraft CS, Wigginton KR, Lowen AC, Marr LC, Rouphael NG, Lakdawala SS. 2024. Influenza virus infection and aerosol shedding kinetics in a controlled human infection model. J Virol 98:e0161224. doi:10.1128/jvi.01612-24 PMC11657674 39589151

[98] Puhach O, Meyer B, Eckerle I. 2022. SARS-CoV-2 viral load and shedding kinetics. Nat Rev Microbiol. doi:10.1038/s41579-022-00822-w PMC9716513 36460930

[99] Janeway CA, et al.. 2001. Principles of innate and adaptive immunity, in Immunobiology: the immune system in health and disease. 5th ed. Garland Science.

[100] Gambadauro A, Galletta F, Li Pomi A, Manti S, Piedimonte G. 2024. Immune response to respiratory viral infections. IJMS 25:6178. doi:10.3390/ijms25116178 PMC11172738 38892370

[101] Okabayashi T, Kojima T, Masaki T, Yokota S-I, Imaizumi T, Tsutsumi H, Himi T, Fujii N, Sawada N. 2011. Type-III interferon, not type-I, is the predominant interferon induced by respiratory viruses in nasal epithelial cells. Virus Res 160:360–366. doi:10.1016/j.virusres.2011.07.011 21816185

[102] Klinkhammer J, Schnepf D, Ye L, Schwaderlapp M, Gad HH, Hartmann R, Garcin D, Mahlakõiv T, Staeheli P. 2018. IFN-λ prevents influenza virus spread from the upper airways to the lungs and limits virus transmission. eLife 7:e33354. doi:10.7554/eLife.33354 PMC5953542 29651984

[103] Jia D, Rahbar R, Chan RWY, Lee SMY, Chan MCW, Wang BX, Baker DP, Sun B, Peiris JSM, Nicholls JM, Fish EN. 2010. Influenza virus non-structural protein 1 (NS1) disrupts interferon signaling. PLoS One 5:e13927. doi:10.1371/journal.pone.0013927 PMC2978095 21085662

[104] Palosaari H, Parisien J-P, Rodriguez JJ, Ulane CM, Horvath CM. 2003. STAT protein interference and suppression of cytokine signal transduction by measles virus V protein. J Virol 77:7635–7644. doi:10.1128/jvi.77.13.7635-7644.2003 PMC164804 12805463

[105] Griffin DE. 2016. The immune response in measles: virus control, clearance and protective immunity. Viruses 8:282. doi:10.3390/v8100282 PMC5086614 27754341

[106] Shivakoti R, Hauer D, Adams RJ, Lin W-H, Duprex WP, de Swart RL, Griffin DE. 2015. Limited *in vivo* production of type I or type III interferon after infection of macaques with vaccine or wild-type strains of measles virus. Journal of Interferon & Cytokine Research 35:292–301. doi:10.1089/jir.2014.0122 PMC4390142 25517681

[107] Yu X, Cheng Y, Shi B, Qian F, Wang F, Liu X, Yang H, Xu Q, Qi T, Zha L, Yuan Z, Ghildyal R. 2008. Measles virus infection in adults induces production of IL-10 and is associated with increased CD4+ CD25+ regulatory T cells. J Immunol 181:7356–7366. doi:10.4049/jimmunol.181.10.7356 18981159

[108] Blanco-Melo D, Nilsson-Payant BE, Liu W-C, Uhl S, Hoagland D, Møller R, Jordan TX, Oishi K, Panis M, Sachs D, Wang TT, Schwartz RE, Lim JK, Albrecht RA, tenOever BR. 2020. Imbalanced host response to SARS-CoV-2 drives development of COVID-19. Cell 181:1036–1045. doi:10.1016/j.cell.2020.04.026 PMC7227586 32416070

[109] Galani I-E, Rovina N, Lampropoulou V, Triantafyllia V, Manioudaki M, Pavlos E, Koukaki E, Fragkou PC, Panou V, Rapti V, Koltsida O, Mentis A, Koulouris N, Tsiodras S, Koutsoukou A, Andreakos E. 2021. Untuned antiviral immunity in COVID-19 revealed by temporal type I/III interferon patterns and flu comparison. Nat Immunol 22:32–40. doi:10.1038/s41590-020-00840-x 33277638

[110] Hadjadj J, Yatim N, Barnabei L, Corneau A, Boussier J, Smith N, Péré H, Charbit B, Bondet V, Chenevier-Gobeaux C, et al.. 2020. Impaired type I interferon activity and inflammatory responses in severe COVID-19 patients. Science 369:718–724. doi:10.1126/science.abc6027 PMC7402632 32661059

[111] Znaidia M, Demeret C, van der Werf S, Komarova AV. 2022. Characterization of SARS-CoV-2 evasion: interferon pathway and therapeutic options. Viruses 14:1247. doi:10.3390/v14061247 PMC9231409 35746718

[112] Croft SN, Walker EJ, Ghildyal R. 2018. Human rhinovirus 3C protease cleaves RIPK1, concurrent with caspase 8 activation. Sci Rep 8. doi:10.1038/s41598-018-19839-4 PMC5785518 29371673

[113] Wark PAB, Johnston SL, Bucchieri F, Powell R, Puddicombe S, Laza-Stanca V, Holgate ST, Davies DE. 2005. Asthmatic bronchial epithelial cells have a deficient innate immune response to infection with rhinovirus. J Exp Med 201:937–947. doi:10.1084/jem.20041901 PMC2213100 15781584

[114] Du X, Yang Y, Xiao G, Yang M, Yuan L, Qin L, He R, Wang L, Wu M, Wu S, Feng J, Xiang Y, Qu X, Liu H, Qin X, Liu C. 2020. Respiratory syncytial virus infection‐induced mucus secretion by down‐regulation of miR‐34b/c‐5p expression in airway epithelial cells. J Cellular Molecular Medi 24:12694–12705. doi:10.1111/jcmm.15845 PMC7687004 32939938

[115] Mata M, Sarrion I, Armengot M, Carda C, Martinez I, Melero JA, Cortijo J. 2012. Respiratory syncytial virus inhibits ciliagenesis in differentiated normal human bronchial epithelial cells: effectiveness of N-acetylcysteine. PLoS ONE 7:e48037. doi:10.1371/journal.pone.0048037 PMC3485262 23118923

[116] Boboltz A, Yang S, Duncan GA. 2023. Engineering *in vitro* models of cystic fibrosis lung disease using neutrophil extracellular trap inspired biomaterials. J Mater Chem B 11:9419–9430. doi:10.1039/d3tb01489d PMC10591795 37701932

[117] Cesta MC, Zippoli M, Marsiglia C, Gavioli EM, Cremonesi G, Khan A, Mantelli F, Allegretti M, Balk R. 2023. Neutrophil activation and neutrophil extracellular traps (NETs) in COVID‐19 ARDS and immunothrombosis. Eur J Immunol 53. doi:10.1002/eji.202250010 PMC9874644 36239164

[118] Saitoh T, Komano J, Saitoh Y, Misawa T, Takahama M, Kozaki T, Uehata T, Iwasaki H, Omori H, Yamaoka S, Yamamoto N, Akira S. 2012. Neutrophil extracellular traps mediate a host defense response to human immunodeficiency virus-1. Cell Host & Microbe 12:109–116. doi:10.1016/j.chom.2012.05.015 22817992

[119] Chen A, Wessler T, Daftari K, Hinton K, Boucher RC, Pickles R, Freeman R, Lai SK, Forest MG. 2022. Modeling insights into SARS-CoV-2 respiratory tract infections prior to immune protection. Biophys J 121:1619–1631. doi:10.1016/j.bpj.2022.04.003 PMC8975607 35378080

[120] Zwart MP, Hemerik L, Cory JS, de Visser JAGM, Bianchi FJJA, Van Oers MM, Vlak JM, Hoekstra RF, Van der Werf W. 2009. An experimental test of the independent action hypothesis in virus–insect pathosystems. Proc R Soc B 276:2233–2242. doi:10.1098/rspb.2009.0064 PMC2677602 19324752

[121] Knight V. 1980. Viruses as agents of airborne contagion. Ann N Y Acad Sci 353:147–156. doi:10.1111/j.1749-6632.1980.tb18917.x 6261640

[122] Alford RH, Kasel JA, Gerone PJ, Knight V. 1966. Human influenza resulting from aerosol inhalation. Exp Biol Med 122:800–804. doi:10.3181/00379727-122-31255 5918954

[123] Han A, Czajkowski LM, Donaldson A, Baus HA, Reed SM, Athota RS, Bristol T, Rosas LA, Cervantes-Medina A, Taubenberger JK, Memoli MJ. 2019. A dose-finding study of a wild-type influenza A(H3N2) virus in a healthy volunteer human challenge model. Clin Infect Dis 69:2082–2090. doi:10.1093/cid/ciz141 PMC6880340 30770534

[124] Couch RB, Cate TR, Douglas RG Jr, Gerone PJ, Knight V. 1966. Effect of route of inoculation on experimental respiratory viral disease in volunteers and evidence for airborne transmission. Bacteriol Rev 30:517–529. doi:10.1128/br.30.3.517-529.1966 PMC378233 5920335

[125] Hendley JO, Wenzel RP, Gwaltney JM. 1973. Transmission of rhinovirus colds by self-inoculation. N Engl J Med 288:1361–1364. doi:10.1056/NEJM197306282882601 4350527

[126] Gwaltney JM, Moskalski PB, Hendley JO. 1978. Hand-to-hand transmission of rhinovirus colds. Ann Intern Med 88:463–467. doi:10.7326/0003-4819-88-4-463 205151

[127] D’Alessio DJ, Meschievitz CK, Peterson JA, Dick CR, Dick EC. 1984. Short-duration exposure and the transmission of rhinoviral colds. J Infect Dis 150:189–194. doi:10.1093/infdis/150.2.189 6088645

[128] Dick EC, Jennings LC, Mink KA, Wartgow CD, Inhorn SL. 1987. Aerosol transmission of rhinovirus colds. J Infect Dis 156:442–448. doi:10.1093/infdis/156.3.442 3039011

[129] Riley EC, Murphy G, Riley RL. 1978. Airborne spread of measles in a suburban elementary school. Am J Epidemiol 107:421–432. doi:10.1093/oxfordjournals.aje.a112560 665658

[130] Samb B, Aaby P, Whittle H, Coll Seck AM, Simondon F. 1993. Protective efficacy of high-titre measles vaccines administered from the age of five months: a community study in rural Senegal. Trans R Soc Trop Med Hyg 87:697–701. doi:10.1016/0035-9203(93)90301-6 8296384

[131] Hou YJ, Okuda K, Edwards CE, Martinez DR, Asakura T, Dinnon KH III, Kato T, Lee RE, Yount BL, Mascenik TM, et al.. 2020. SARS-CoV-2 reverse genetics reveals a variable infection gradient in the respiratory tract. Cell 182:429–446. doi:10.1016/j.cell.2020.05.042 PMC7250779 32526206

[132] Miller SL, Nazaroff WW, Jimenez JL, Boerstra A, Buonanno G, Dancer SJ, Kurnitski J, Marr LC, Morawska L, Noakes C. 2021. Transmission of SARS‐CoV‐2 by inhalation of respiratory aerosol in the Skagit Valley Chorale superspreading event. Indoor Air 31:314–323. doi:10.1111/ina.12751 PMC7537089 32979298

[133] Groma V, Kugler Sz, Farkas Á, Füri P, Madas B, Nagy A, Erdélyi T, Horváth A, Müller V, Szántó-Egész R, Micsinai A, Gálffy G, Osán J. 2023. Size distribution and relationship of airborne SARS-CoV-2 RNA to indoor aerosol in hospital ward environments. Sci Rep 13:1. doi:10.1038/s41598-023-30702-z PMC9980870 36864124

[134] Alsved M, Nygren D, Thuresson S, Fraenkel C-J, Medstrand P, Löndahl J. 2023. Size distribution of exhaled aerosol particles containing SARS-CoV-2 RNA. Infect Dis (Lond) 55:158–163. doi:10.1080/23744235.2022.2140822 36331347

[135] Ryan KA, Bewley KR, Fotheringham SA, Slack GS, Brown P, Hall Y, Wand NI, Marriott AC, Cavell BE, Tree JA, et al.. 2021. Dose-dependent response to infection with SARS-CoV-2 in the ferret model and evidence of protective immunity. Nat Commun 12:2021–01 doi:10.1038/s41467-020-20439-y PMC7782478 33398055

[136] Wölfel R, Corman VM, Guggemos W, Seilmaier M, Zange S, Müller MA, Niemeyer D, Jones TC, Vollmar P, Rothe C, Hoelscher M, Bleicker T, Brünink S, Schneider J, Ehmann R, Zwirglmaier K, Drosten C, Wendtner C. 2020. Virological assessment of hospitalized patients with COVID-2019. Nature 581:465–469. doi:10.1038/s41586-020-2196-x 32235945

[137] Sarkar JK, Mitra AC, Mukherjee MK, De SK. 1973. Virus excretion in smallpox. 2. Excretion in the throats of household contacts. Bull World Health Organ 48:523–527. PMC2482939 4359679

[138] Sarkar JK, Mitra AC, Mukherjee MK, De SK, Mazumdar DG. 1973. Virus excretion in smallpox. 1. Excretion in the throat, urine, and conjunctiva of patients. Bull World Health Organ 48:517–522. PMC2482928 4359678

[139] Stadnytskyi V, Bax CE, Bax A, Anfinrud P. 2020. The airborne lifetime of small speech droplets and their potential importance in SARS-CoV-2 transmission. Proc Natl Acad Sci USA 117:11875–11877. doi:10.1073/pnas.2006874117 PMC7275719 32404416

[140] Rodriguez-Boulan E, Macara IG. 2014. Organization and execution of the epithelial polarity programme. Nat Rev Mol Cell Biol 15:225–242. doi:10.1038/nrm3775 PMC4211427 24651541

[141] Chapuy-Regaud S, Allioux C, Capelli N, Migueres M, Lhomme S, Izopet J. 2022. Vectorial release of human RNA viruses from epithelial cells. Viruses 14:2022–01 doi:10.3390/v14020231 PMC8875463 35215825

[142] Tseng C-TK, Tseng J, Perrone L, Worthy M, Popov V, Peters CJ. 2005. Apical entry and release of severe acute respiratory syndrome-associated coronavirus in polarized calu-3 lung epithelial cells. J Virol 79:9470–9479. doi:10.1128/JVI.79.15.9470-9479.2005 PMC1181546 16014910

[143] Wu N-H, Yang W, Beineke A, Dijkman R, Matrosovich M, Baumgärtner W, Thiel V, Valentin-Weigand P, Meng F, Herrler G. 2016. The differentiated airway epithelium infected by influenza viruses maintains the barrier function despite a dramatic loss of ciliated cells. Sci Rep 6:39668. doi:10.1038/srep39668 PMC5177954 28004801

[144] Tao X, Hill TE, Morimoto C, Peters CJ, Ksiazek TG, Tseng C-TK. 2013. Bilateral entry and release of Middle East respiratory syndrome coronavirus induces profound apoptosis of human bronchial epithelial cells. J Virol 87:9953–9958. doi:10.1128/JVI.01562-13 PMC3754134 23824802

[145] Zhu N, Wang W, Liu Z, Liang C, Wang W, Ye F, Huang B, Zhao L, Wang H, Zhou W, Deng Y, Mao L, Su C, Qiang G, Jiang T, Zhao J, Wu G, Song J, Tan W. 2020. Morphogenesis and cytopathic effect of SARS-CoV-2 infection in human airway epithelial cells. Nat Commun 11:3910. doi:10.1038/s41467-020-17796-z PMC7413383 32764693

[146] Wu C-T, Lidsky PV, Xiao Y, Cheng R, Lee IT, Nakayama T, Jiang S, He W, Demeter J, Knight MG, Turn RE, Rojas-Hernandez LS, Ye C, Chiem K, Shon J, Martinez-Sobrido L, Bertozzi CR, Nolan GP, Nayak JV, Milla C, Andino R, Jackson PK. 2023. SARS-CoV-2 replication in airway epithelia requires motile cilia and microvillar reprogramming. Cell 186:112–130. doi:10.1016/j.cell.2022.11.030 PMC9715480 36580912

[147] Tucker SP, Compans RW. 1993. Virus infection of polarized epithelial cells. Adv Virus Res 42:187–247. doi:10.1016/s0065-3527(08)60086-x PMC7130709 8430520

[148] Hippee CE, Singh BK, Thurman AL, Cooney AL, Pezzulo AA, Cattaneo R, Sinn PL. 2021. Measles virus exits human airway epithelia within dislodged metabolically active infectious centers. PLOS Pathog 17:e1009458. doi:10.1371/journal.ppat.1009458 PMC8384213 34383863

[149] Ghosh S, Dellibovi-Ragheb TA, Kerviel A, Pak E, Qiu Q, Fisher M, Takvorian PM, Bleck C, Hsu VW, Fehr AR, Perlman S, Achar SR, Straus MR, Whittaker GR, de Haan CAM, Kehrl J, Altan-Bonnet G, Altan-Bonnet N. 2020. β-coronaviruses use lysosomes for egress instead of the biosynthetic secretory pathway. Cell 183:1520–1535. doi:10.1016/j.cell.2020.10.039 PMC7590812 33157038

[150] Zhang L, Peeples ME, Boucher RC, Collins PL, Pickles RJ. 2002. Respiratory syncytial virus infection of human airway epithelial cells is polarized, specific to ciliated cells, and without obvious cytopathology. J Virol 76:5654–5666. doi:10.1128/JVI.76.11.5654-5666.2002 PMC137037 11991994

[151] Zeng C, Evans JP, King T, Zheng Y-M, Oltz EM, Whelan SPJ, Saif LJ, Peeples ME, Liu S-L. 2022. SARS-CoV-2 spreads through cell-to-cell transmission. Proc Natl Acad Sci USA 119. doi:10.1073/pnas.2111400119 PMC8740724 34937699

[152] Gerl MJ, Sampaio JL, Urban S, Kalvodova L, Verbavatz J-M, Binnington B, Lindemann D, Lingwood CA, Shevchenko A, Schroeder C, Simons K. 2012. Quantitative analysis of the lipidomes of the influenza virus envelope and MDCK cell apical membrane. J Cell Biol 196:213–221. doi:10.1083/jcb.201108175 PMC3265945 22249292

[153] Lorizate M, Krausslich H-G. 2011. Role of lipids in virus replication. Cold Spring Harb Perspect Biol 3:a004820–a004820. doi:10.1101/cshperspect.a004820 PMC3179339 21628428

[154] Brémaud E, Favard C, Muriaux D. 2022. Deciphering the assembly of enveloped viruses using model lipid membranes. Membranes (Basel) 12:441. doi:10.3390/membranes12050441 PMC9142974 35629766

[155] de Wit E, Rasmussen AL, Falzarano D, Bushmaker T, Feldmann F, Brining DL, Fischer ER, Martellaro C, Okumura A, Chang J, Scott D, Benecke AG, Katze MG, Feldmann H, Munster VJ. 2013. Middle East respiratory syndrome coronavirus (MERS-CoV) causes transient lower respiratory tract infection in rhesus macaques. Proc Natl Acad Sci USA 110:16598–16603. doi:10.1073/pnas.1310744110 PMC3799368 24062443

[156] Cevik M, Tate M, Lloyd O, Maraolo AE, Schafers J, Ho A. 2020. SARS-CoV-2, SARS-CoV-1 and MERS-CoV viral load dynamics, duration of viral shedding and infectiousness: a living systematic review and meta-analysis. SSRN Journal. doi:10.2139/ssrn.3677918 PMC7837230 33521734

[157] Peiris JSM, Lai ST, Poon LLM, Guan Y, Yam LYC, Lim W, Nicholls J, Yee WKS, Yan WW, Cheung MT, Cheng VCC, Chan KH, Tsang DNC, Yung RWH, Ng TK, Yuen KY. 2003. Coronavirus as a possible cause of severe acute respiratory syndrome. The Lancet 361:1319–1325. doi:10.1016/S0140-6736(03)13077-2 PMC7112372 12711465

[158] Widagdo W, Raj VS, Schipper D, Kolijn K, van Leenders GJLH, Bosch BJ, Bensaid A, Segalés J, Baumgärtner W, Osterhaus ADME, Koopmans MP, van den Brand JMA, Haagmans BL. 2016. Differential expression of the Middle East respiratory syndrome coronavirus receptor in the upper respiratory tracts of humans and dromedary camels. J Virol 90:4838–4842. doi:10.1128/JVI.02994-15 PMC4836314 26889022

[159] Richard M, van den Brand JMA, Bestebroer TM, Lexmond P, de Meulder D, Fouchier RAM, Lowen AC, Herfst S. 2020. Influenza A viruses are transmitted via the air from the nasal respiratory epithelium of ferrets. Nat Commun 11. doi:10.1038/s41467-020-14626-0 PMC7005743 32034144

[160] Lakdawala SS, Jayaraman A, Halpin RA, Lamirande EW, Shih AR, Stockwell TB, Lin X, Simenauer A, Hanson CT, Vogel L, Paskel M, Minai M, Moore I, Orandle M, Das SR, Wentworth DE, Sasisekharan R, Subbarao K. 2015. The soft palate is an important site of adaptation for transmissible influenza viruses. Nature 526:122–125. doi:10.1038/nature15379 PMC4592815 26416728

[161] Trende R, Darling TL, Gan T, Wang D, Boon ACM. 2025. Barcoded SARS-CoV-2 viruses define the impact of duration and route of exposure on the transmission bottleneck in a hamster model. Sci Adv 11:eads2927. doi:10.1126/sciadv.ads2927 PMC11778309 39813353

[162] Burke CW, Bridges O, Brown S, Rahija R, Russell CJ. 2013. Mode of parainfluenza virus transmission determines the dynamics of primary infection and protection from reinfection. PLoS Pathog 9:e1003786. doi:10.1371/journal.ppat.1003786 PMC3836739 24278024

[163] Hall CB, Douglas RG Jr. 1981. Modes of transmission of respiratory syncytial virus. J Pediatr 99:100–103. doi:10.1016/S0022-3476(81)80969-9 7252646

[164] Huong TN, Yan Y, Jumat MR, Lui J, Tan BH, Wang DY, Sugrue RJ. 2018. A sustained antiviral host response in respiratory syncytial virus infected human nasal epithelium does not prevent progeny virus production. Virology 521:20–32. doi:10.1016/j.virol.2018.05.012 29870884

[165] Johnson GR, Morawska L, Ristovski ZD, Hargreaves M, Mengersen K, Chao CYH, Wan MP, Li Y, Xie X, Katoshevski D, Corbett S. 2011. Modality of human expired aerosol size distributions. J Aerosol Sci 42:839–851. doi:10.1016/j.jaerosci.2011.07.009 PMC7126899 32287373

[166] Almstrand A-C, Bake B, Ljungström E, Larsson P, Bredberg A, Mirgorodskaya E, Olin A-C. 2010. Effect of airway opening on production of exhaled particles. J Appl Physiol 108:584–588. doi:10.1152/japplphysiol.00873.2009 20056850

[167] Xie X, Li Y, Sun H, Liu L. 2009. Exhaled droplets due to talking and coughing. J R Soc Interface 6 Suppl 6:S703–14. doi:10.1098/rsif.2009.0388.focus PMC2843952 19812073

[168] van Mersbergen M, Marchetta J, Foti D, Pillow E, Dasgupta A, Cain C, Morvant S. 2025. Comparison of aerosol emissions during specific speech tasks. J Voice 39:1276–1285. doi:10.1016/j.jvoice.2023.05.004 37423794

[169] Abkarian M, Stone HA. 2020. Stretching and break-up of saliva filaments during speech: a route for pathogen aerosolization and its potential mitigation. Phys Rev Fluids 5:10. doi:10.1103/PhysRevFluids.5.102301

[170] Alsved M, Matamis A, Bohlin R, Richter M, Bengtsson P-E, Fraenkel C-J, Medstrand P, Löndahl J. 2020. Exhaled respiratory particles during singing and talking. Aerosol Science and Technology 54:1245–1248. doi:10.1080/02786826.2020.1812502

[171] Asadi S, Wexler AS, Cappa CD, Barreda S, Bouvier NM, Ristenpart WD. 2019. Aerosol emission and superemission during human speech increase with voice loudness. Sci Rep 9:2348. doi:10.1038/s41598-019-38808-z PMC6382806 30787335

[172] Zhu S, Kato S, Yang J-H. 2006. Study on transport characteristics of saliva droplets produced by coughing in a calm indoor environment. Build Environ 41:1691–1702. doi:10.1016/j.buildenv.2005.06.024

[173] Han M, Ooka R, Kikumoto H, Oh W, Bu Y, Hu S. 2021. Measurements of exhaled airflow velocity through human coughs using particle image velocimetry. Build Environ 202:108020. doi:10.1016/j.buildenv.2021.108020 PMC8188782 34127875

[174] Scharfman BE, Techet AH, Bush JWM, Bourouiba L. 2016. Visualization of sneeze ejecta: steps of fluid fragmentation leading to respiratory droplets. Exp Fluids 57:24. doi:10.1007/s00348-015-2078-4 PMC7088075 32214638

[175] Bourouiba L, Dehandschoewercker E, Bush JWM. 2014. Violent expiratory events: on coughing and sneezing. J Fluid Mech 745:537–563. doi:10.1017/jfm.2014.88

[176] Li M, Saade Y, Zaleski S, Sen U, Kant P, Lohse D. 2025. Viscoelasticity reduces the droplet size in mucosalivary film fragmentation during intense respiratory events. Phys Rev Fluids 10. doi:10.1103/zlgy-36dk

[177] Wang CC, Prather KA, Sznitman J, Jimenez JL, Lakdawala SS, Tufekci Z, Marr LC. 2021. Airborne transmission of respiratory viruses. Science 373:eabd9149. doi:10.1126/science.abd9149 PMC8721651 34446582

[178] Alsved M, Nygren D, Thuresson S, Fraenkel C-J, Medstrand P, Löndahl J. 2023. Size distribution of exhaled aerosol particles containing SARS-CoV-2 RNA. Infectious Diseases 55:158–163. doi:10.1080/23744235.2022.2140822 36331347

[179] Lau LLH, Cowling BJ, Fang VJ, Chan K, Lau EHY, Lipsitch M, Cheng CKY, Houck PM, Uyeki TM, Peiris JSM, Leung GM. 2010. Viral shedding and clinical illness in naturally acquired influenza virus infections. J INFECT DIS 201:1509–1516. doi:10.1086/652241 PMC3060408 20377412

[180] Zou L, Ruan F, Huang M, Liang L, Huang H, Hong Z, Yu J, Kang M, Song Y, Xia J, Guo Q, Song T, He J, Yen H-L, Peiris M, Wu J. 2020. SARS-CoV-2 viral load in upper respiratory specimens of infected patients. N Engl J Med 382:1177–1179. doi:10.1056/NEJMc2001737 PMC7121626 32074444

[181] He X, Lau EHY, Wu P, Deng X, Wang J, Hao X, Lau YC, Wong JY, Guan Y, Tan X, Mo X, Chen Y, Liao B, Chen W, Hu F, Zhang Q, Zhong M, Wu Y, Zhao L, Zhang F, Cowling BJ, Li F, Leung GM. 2020. Temporal dynamics in viral shedding and transmissibility of COVID-19. Nat Med 26:672–675. doi:10.1038/s41591-020-0869-5 32296168

[182] Tosheva II, Filaire F, Rijnink WF, de Meulder D, van Kekem B, Bestebroer TM, Funk M, Spronken MI, Cáceres CJ, Perez DR, Richard M, Koopmans MPG, Fraaij PLA, Fouchier RAM, Herfst S. 2025. Influenza A(H5N1) shedding in air corresponds to transmissibility in mammals. Nat Microbiol 10:14–19. doi:10.1038/s41564-024-01885-6 PMC11726459 39623068

[183] Okiro EA, White LJ, Ngama M, Cane PA, Medley GF, Nokes DJ. 2010. Duration of shedding of respiratory syncytial virus in a community study of Kenyan children. BMC Infect Dis 10:15. doi:10.1186/1471-2334-10-15 PMC2822777 20096106

[184] Linster M, Do LAH, Minh NNQ, Chen Y, Zhe Z, Tuan TA, Tuan HM, Su YCF, van Doorn HR, Moorthy M, Smith GJD. 2018. Clinical and molecular epidemiology of human parainfluenza viruses 1-4 in children from Viet Nam. Sci Rep 8:6833. doi:10.1038/s41598-018-24767-4 PMC5931535 29717150

[185] French AJ, Longest AK, Pan J, Vikesland PJ, Duggal NK, Marr LC, Lakdawala SS. 2023. Environmental stability of enveloped viruses is impacted by initial volume and evaporation kinetics of droplets. mBio 14:e0345222. doi:10.1128/mbio.03452-22 PMC10128059 37036343

[186] HIATT CW. 1964. Kinetics of the inactivation of viruses. Bacteriol Rev 28:150–163. doi:10.1128/br.28.2.150-163.1964 PMC441218 14172021

[187] Merhi T, Atasi O, Coetsier C, Lalanne B, Roger K. 2022. Assessing suspension and infectivity times of virus-loaded aerosols involved in airborne transmission. Proc Natl Acad Sci USA 119:32. doi:10.1073/pnas.2204593119 PMC9371747 35930663

[188] Huynh E, Olinger A, Woolley D, Kohli RK, Choczynski JM, Davies JF, Lin K, Marr LC, Davis RD. 2022. Evidence for a semisolid phase state of aerosols and droplets relevant to the airborne and surface survival of pathogens. Proc Natl Acad Sci USA 119:e2109750119. doi:10.1073/pnas.2109750119 PMC8794803 35064080

[189] Vejerano EP, Marr LC. 2018. Physico-chemical characteristics of evaporating respiratory fluid droplets. J R Soc Interface 15:20170939. doi:10.1098/rsif.2017.0939 PMC5832737 29491178

[190] David SC, Vadas O, Glas I, Schaub A, Luo B, D’angelo G, Montoya JP, Bluvshtein N, Hugentobler W, Klein LK, Motos G, Pohl M, Violaki K, Nenes A, Krieger UK, Stertz S, Peter T, Kohn T. 2023. Inactivation mechanisms of influenza A virus under pH conditions encountered in aerosol particles as revealed by whole-virus HDX-MS. mSphere 8:e0022623. doi:10.1128/msphere.00226-23 PMC10597348 37594288

[191] Luo B, Schaub A, Glas I, Klein LK, David SC, Bluvshtein N, Violaki K, Motos G, Pohl MO, Hugentobler W, Nenes A, Krieger UK, Stertz S, Peter T, Kohn T. 2023. Expiratory aerosol pH: the overlooked driver of airborne virus inactivation. Environ Sci Technol 57:486–497. doi:10.1021/acs.est.2c05777 PMC9835828 36537693

[192] Firquet S, Beaujard S, Lobert P-E, Sané F, Caloone D, Izard D, Hober D. 2015. Survival of enveloped and non-enveloped viruses on inanimate surfaces. Microbes Environ 30:140–144. doi:10.1264/jsme2.ME14145 PMC4462923 25843687

[193] Longest AK, Rockey NC, Lakdawala SS, Marr LC. 2024. Review of factors affecting virus inactivation in aerosols and droplets. J R Soc Interface 21:18. doi:10.1098/rsif.2024.0018 PMC11285516 38920060

[194] Oswin HP, Haddrell AE, Otero-Fernandez M, Mann JFS, Cogan TA, Hilditch TG, Tian J, Hardy DA, Hill DJ, Finn A, Davidson AD, Reid JP. 2022. The dynamics of SARS-CoV-2 infectivity with changes in aerosol microenvironment. Proc Natl Acad Sci USA 119:27. doi:10.1073/pnas.2200109119 PMC9271203 35763573

[195] Haddrell A, Otero-Fernandez M, Oswin H, Cogan T, Bazire J, Tian J, Alexander R, Mann JFS, Hill D, Finn A, Davidson AD, Reid JP. 2023. Differences in airborne stability of SARS-CoV-2 variants of concern is impacted by alkalinity of surrogates of respiratory aerosol. J R Soc Interface 20:20230062. doi:10.1098/rsif.2023.0062 PMC10282576 37340783

[196] Alexander RW, Tian J, Haddrell AE, Oswin HP, Neal E, Hardy DA, Otero-Fernandez M, Mann JFS, Cogan TA, Finn A, Davidson AD, Hill DJ, Reid JP. 2022. Mucin transiently sustains coronavirus infectivity through heterogenous changes in phase morphology of evaporating aerosol. Viruses 14:1856. doi:10.3390/v14091856 PMC9503081 36146663

[197] Pan J, Duggal NK, Lakdawala SS, Rockey NC, Marr LC. 2025. Mucin colocalizes with influenza virus and preserves infectivity in deposited model respiratory droplets. Environ Sci Technol 59:2192–2200. doi:10.1021/acs.est.4c10886 PMC12875289 39823314

[198] Kormuth KA, Lin K, Prussin AJ 2nd, Vejerano EP, Tiwari AJ, Cox SS, Myerburg MM, Lakdawala SS, Marr LC. 2018. Influenza virus infectivity is retained in aerosols and droplets independent of relative humidity. J Infect Dis 218:739–747. doi:10.1093/infdis/jiy221 PMC6057527 29878137

[199] Dabisch PA, Wood SP, Holland BP, Boydston JA, Beck KE, Green B, Biryukov J. 2022. Comparison of the survival of different isolates of SARS-CoV-2 in evaporating aerosols. Aerosol Sci Technol 56:1146–1155. doi:10.1080/02786826.2022.2128712 PMC10698713 38075547

[200] Morris DH, Yinda KC, Gamble A, Rossine FW, Huang Q, Bushmaker T, Fischer RJ, Matson MJ, Van Doremalen N, Vikesland PJ, Marr LC, Munster VJ, Lloyd-Smith JO. 2021. Mechanistic theory predicts the effects of temperature and humidity on inactivation of SARS-CoV-2 and other enveloped viruses. eLife 10:e65902. doi:10.7554/eLife.65902 PMC8277363 33904403

[201] Morawska L, Li Y, Salthammer T. 2024. Lessons from the COVID-19 pandemic for ventilation and indoor air quality. Science 385:396–401. doi:10.1126/science.adp2241 39052782

[202] McKee CD, Yu EX, Garcia A, Jackson J, Koyuncu A, Rose S, Azman AS, Lobner K, Sacks E, Van Kerkhove MD, Gurley ES. 2024. Superspreading of SARS-CoV-2: a systematic review and meta-analysis of event attack rates and individual transmission patterns. Epidemiol Infect 152:e121. doi:10.1017/S0950268824000955 PMC11488467 39377138

[203] Hope K, Boyd R, Conaty S, Maywood P, South Western Sydney and Sydney Local Health Districts Public Health Unit, New South Wales, Australia. 2012. Measles transmission in health care waiting rooms: implications for public health response. Western Pac Surveill Response J 3:33–38. doi:10.5365/WPSAR.2012.3.3.009 PMC3729094 23908937

[204] van Doremalen N, Bushmaker T, Munster VJ. 2013. Stability of Middle East respiratory syndrome coronavirus (MERS-CoV) under different environmental conditions. Euro Surveill 18:20590. doi:10.2807/1560-7917.ES2013.18.38.20590 24084338

[205] Harper GJ. 1961. Airborne micro-organisms: survival tests with four viruses. Epidemiol Infect 59:479–486. doi:10.1017/S0022172400039176 PMC2134455 13904777

[206] Marr LC, Tang JW, Van Mullekom J, Lakdawala SS. 2019. Mechanistic insights into the effect of humidity on airborne influenza virus survival, transmission and incidence. J R Soc Interface 16:20180298. doi:10.1098/rsif.2018.0298 PMC6364647 30958176

[207] Schaffer FL, Soergel ME, Straube DC. 1976. Survival of airborne influenza virus: effects of propagating host, relative humidity, and composition of spray fluids. Arch Virol 51:263–273. doi:10.1007/BF01317930 987765

[208] Smither SJ, Eastaugh LS, Findlay JS, Lever MS. 2020. Experimental aerosol survival of SARS-CoV-2 in artificial saliva and tissue culture media at medium and high humidity. Emerging Microbes & Infections 9:1415–1417. doi:10.1080/22221751.2020.1777906 PMC7473326 32496967

[209] Schuit M, Ratnesar-Shumate S, Yolitz J, Williams G, Weaver W, Green B, Miller D, Krause M, Beck K, Wood S, Holland B, Bohannon J, Freeburger D, Hooper I, Biryukov J, Altamura LA, Wahl V, Hevey M, Dabisch P. 2020. Airborne SARS-CoV-2 is rapidly inactivated by simulated sunlight. J Infect Dis 222:564–571. doi:10.1093/infdis/jiaa334 PMC7313838 32525979

[210] DE JONG JG, WINKLER KC. 1964. Survival of measles virus in air. Nature 201:1054–1055. doi:10.1038/2011054a0 14191599

[211] Donaldson AI. 1973. The influence of relative humidity on the stability of foot-and-mouth disease virus in aerosols from milk and faecal slurry. Res Vet Sci 15:96–101. doi:10.1016/S0034-5288(18)33859-1 4360318

[212] Yang W, Marr LC. 2012. Mechanisms by which ambient humidity may affect viruses in aerosols. Appl Environ Microbiol 78:6781–6788. doi:10.1128/AEM.01658-12 PMC3457514 22820337

[213] Motos G, Schaub A, David SC, Costa L, Terrettaz C, Kaltsonoudis C, Glas I, Klein LK, Bluvshtein N, Luo B, Violaki K, Pohl MO, Hugentobler W, Krieger UK, Pandis SN, Stertz S, Peter T, Kohn T, Nenes A. 2024. Dependence of aerosol-borne influenza A virus infectivity on relative humidity and aerosol composition. Front Microbiol 15:1484992. doi:10.3389/fmicb.2024.1484992 PMC11521868 39479211

[214] Pereira de Sousa I, Steiner C, Schmutzler M, Wilcox MD, Veldhuis GJ, Pearson JP, Huck CW, Salvenmoser W, Bernkop-Schnürch A. 2015. Mucus permeating carriers: formulation and characterization of highly densely charged nanoparticles. Eur J Pharm Biopharm 97:273–279. doi:10.1016/j.ejpb.2014.12.024 25576256

[215] Kolbe T, Gaspard P, Mognetti BM. 2025. Understanding influenza A virus particles detaching from reconstructed cell surfaces. Nano Lett 25:15340–15345. doi:10.1021/acs.nanolett.5c04036 41071005

[216] Schuster BS, Suk JS, Woodworth GF, Hanes J. 2013. Nanoparticle diffusion in respiratory mucus from humans without lung disease. Biomaterials 34:3439–3446. doi:10.1016/j.biomaterials.2013.01.064 PMC3590854 23384790

[217] Barnard KN, Alford-Lawrence BK, Buchholz DW, Wasik BR, LaClair JR, Yu H, Honce R, Ruhl S, Pajic P, Daugherity EK, Chen X, Schultz-Cherry SL, Aguilar HC, Varki A, Parrish CR. 2020. Modified sialic acids on mucus and erythrocytes inhibit influenza A virus hemagglutinin and neuraminidase functions. J Virol 94:e01567-19. doi:10.1128/JVI.01567-19 PMC7163148 32051275

[218] de Vries E, Du W, Guo H, de Haan CAM. 2020. Influenza A virus hemagglutinin–neuraminidase–receptor balance: preserving virus motility. Trends Microbiol 28:57–67. doi:10.1016/j.tim.2019.08.010 PMC7172302 31629602

[219] Bouvier NM. 2015. Animal models for influenza virus transmission studies: a historical perspective. Curr Opin Virol 13:101–108. doi:10.1016/j.coviro.2015.06.002 PMC4550509 26126082

[220] Chu H, Chan JF-W, Yuen K-Y. 2022. Animal models in SARS-CoV-2 research. Nat Methods 19:392–394. doi:10.1038/s41592-022-01447-w 35396468

[221] Kistler K, Bedford T. 2025. Seasonal influenza viruses show distinct adaptive dynamics during growth in chicken eggs. Mol Biol Evol 42:msaf227. doi:10.1093/molbev/msaf227 PMC12492274 40974095

[222] King T, Mejias A, Ramilo O, Peeples ME. 2021. The larger attachment glycoprotein of respiratory syncytial virus produced in primary human bronchial epithelial cultures reduces infectivity for cell lines. PLOS Pathog 17:e1009469. doi:10.1371/journal.ppat.1009469 PMC8057581 33831114

[223] Ito K, Daly L, Coates M. 2023. An impact of age on respiratory syncytial virus infection in air-liquid-interface culture bronchial epithelium. Front Med 10:10. doi:10.3389/fmed.2023.1144050 PMC10043235 36999069

[224] Gindele JA, Kiechle T, Benediktus K, Birk G, Brendel M, Heinemann F, Wohnhaas CT, LeBlanc M, Zhang H, Strulovici-Barel Y, Crystal RG, Thomas MJ, Stierstorfer B, Quast K, Schymeinsky J. 2020. Intermittent exposure to whole cigarette smoke alters the differentiation of primary small airway epithelial cells in the air-liquid interface culture. Sci Rep 10:1. doi:10.1038/s41598-020-63345-5 PMC7148343 32277131

[225] Lutsch CT, Feng L, Gómez Hohn A, Brandt L, Tamm S, Janciauskiene S, Stanke F, Jonigk D, Dittrich A-M, Braubach P. 2024. A fast scoring of human primary respiratory epithelia grown at air-liquid interface (ALI) to assess epithelial morphology in research and personalized medicine settings. J Pers Med 14:109. doi:10.3390/jpm14010109 PMC10817428 38248810

[226] Gastañaduy PA, Funk S, Lopman BA, Rota PA, Gambhir M, Grenfell B, Paul P. 2020. Factors associated with measles transmission in the United States during the postelimination era. JAMA Pediatr 174:56. doi:10.1001/jamapediatrics.2019.4357 PMC6865326 31738391

[227] Cauchemez S, Donnelly CA, Reed C, Ghani AC, Fraser C, Kent CK, Finelli L, Ferguson NM. 2009. Household transmission of 2009 pandemic influenza A (H1N1) virus in the United States. N Engl J Med 361:2619–2627. doi:10.1056/NEJMoa0905498 PMC3840270 20042753

[228] Munywoki PK, Koech DC, Agoti CN, Lewa C, Cane PA, Medley GF, Nokes DJ. 2014. The source of respiratory syncytial virus infection in infants: a household cohort study in rural Kenya. J Infect Dis 209:1685–1692. doi:10.1093/infdis/jit828 PMC4017365 24367040

[229] Heikkinen T, Valkonen H, Waris M, Ruuskanen O. 2015. Transmission of respiratory syncytial virus infection within families. Open Forum Infect Dis 2:fu118. doi:10.1093/ofid/ofu118 PMC4396434 25884006

[230] Chan T-C, Hu T-H, Hwang J-S. 2018. Estimating the risk of influenza-like illness transmission through social contacts: web-based participatory cohort study. JMIR Public Health Surveill 4:e40. doi:10.2196/publichealth.8874 PMC5913573 29631987

[231] Jacoby P, Glass K, Moore HC. 2017. Characterizing the risk of respiratory syncytial virus in infants with older siblings: a population-based birth cohort study. Epidemiol Infect 145:266–271. doi:10.1017/S0950268816002545 PMC9507629 27821195

[232] Miller E, Waight PA, Andrews NJ, McOwat K, Brown KE, Höschler K, Ijaz S, Letley L, Haskins D, Sinnathamby M, Cuthbertson H, Hallis B, Parimalanathan V, de Lusignan S, Lopez-Bernal J. 2021. Transmission of SARS-CoV-2 in the household setting: a prospective cohort study in children and adults in England. J Infect 83:483–489. doi:10.1016/j.jinf.2021.07.037 PMC8325949 34348116

[233] Li F, Li Y-Y, Liu M-J, Fang L-Q, Dean NE, Wong GWK, Yang X-B, Longini I, Halloran ME, Wang H-J, Liu P-L, Pang Y-H, Yan Y-Q, Liu S, Xia W, Lu X-X, Liu Q, Yang Y, Xu S-Q. 2021. Household transmission of SARS-CoV-2 and risk factors for susceptibility and infectivity in Wuhan: a retrospective observational study. Lancet Infect Dis 21:617–628. doi:10.1016/S1473-3099(20)30981-6 PMC7833912 33476567

[234] Madewell ZJ, Yang Y, Longini IM Jr, Halloran ME, Dean NE. 2021. Factors associated with household transmission of SARS-CoV-2: an updated systematic review and meta-analysis. JAMA Netw Open 4:e2122240. doi:10.1001/jamanetworkopen.2021.22240 PMC8397928 34448865

[235] Klein SL, Flanagan KL. 2016. Sex differences in immune responses. Nat Rev Immunol 16:626–638. doi:10.1038/nri.2016.90 27546235

[236] Port JR, Yinda CK, Ruckel C, Schulz JE, Smith BJ, Shaia CI, Munster VJ. 2024. Augmentation of omicron BA.1 pathogenicity in hamsters using intratracheal inoculation. Npj Viruses 2:3. doi:10.1038/s44298-023-00012-2 PMC11702663 40295685

[237] Bai Y, He Q, Yang J, Lu S, Mao Q, Gao F, Bian L, Zhang J, An C, Liu J, Wu X, Yu W, Wang Z, Peng X, Wang J, Liang Z, Xu M. 2022. B.1.351 SARS-CoV-2 variant exhibits higher virulence but less viral shedding than that of the ancestral strain in young Nonhuman primates. Microbiol Spectr 10:e0226322. doi:10.1128/spectrum.02263-22 PMC9603226 36069561

[238] Tran V, Moser LA, Poole DS, Mehle A. 2013. Highly sensitive real-time *in vivo* imaging of an influenza reporter virus reveals dynamics of replication and spread. J Virol 87:13321–13329. doi:10.1128/JVI.02381-13 PMC3838222 24089552

[239] Manicassamy B, Manicassamy S, Belicha-Villanueva A, Pisanelli G, Pulendran B, García-Sastre A. 2010. Analysis of *in vivo* dynamics of influenza virus infection in mice using a GFP reporter virus. Proc Natl Acad Sci USA 107:11531–11536. doi:10.1073/pnas.0914994107 PMC2895123 20534532

[240] Varble A, Albrecht RA, Backes S, Crumiller M, Bouvier NM, Sachs D, García-Sastre A, tenOever BR. 2014. Influenza A virus transmission bottlenecks are defined by infection route and recipient host. Cell Host & Microbe 16:691–700. doi:10.1016/j.chom.2014.09.020 PMC4272616 25456074

[241] Osterholm MT, Moore KA, Kelley NS, Brosseau LM, Wong G, Murphy FA, Peters CJ, LeDuc JW, Russell PK, Van Herp M, Kapetshi J, Muyembe J-JT, Ilunga BK, Strong JE, Grolla A, Wolz A, Kargbo B, Kargbo DK, Formenty P, Sanders DA, Kobinger GP. 2015. Transmission of ebola viruses: what we know and what we do not know. mBio 6. doi:10.1128/mBio.00137-15 PMC4358015 25698835

[242] Wang Y, Wang X, Doğan T, Sam-Agudu NA, Al-Tawfiq JA, Pan Q. 2025. Mpox: disease manifestations and therapeutic development. J Virol 99. doi:10.1128/jvi.00152-25 PMC12459235 40787989

[243] Nie Y, Wang P, Shi X, Wang G, Chen J, Zheng A, Wang W, Wang Z, Qu X, Luo M, Tan L, Song X, Yin X, Chen J, Ding M, Deng H. 2004. Highly infectious SARS-CoV pseudotyped virus reveals the cell tropism and its correlation with receptor expression. Biochem Biophys Res Commun 321:994–1000. doi:10.1016/j.bbrc.2004.07.060 PMC7092805 15358126

[244] Rockey NC, Le Sage V, Marr LC, Lakdawala SS. 2024. Seasonal influenza viruses decay more rapidly at intermediate humidity in droplets containing saliva compared to respiratory mucus. Appl Environ Microbiol 90:e0201023. doi:10.1128/aem.02010-23 PMC10880610 38193683

[245] Cao X, Coyle JP, Xiong R, Wang Y, Heflich RH, Ren B, Gwinn WM, Hayden P, Rojanasakul L. 2021. Invited review: human air-liquid-interface organotypic airway tissue models derived from primary tracheobronchial epithelial cells—overview and perspectives. In Vitro CellDevBiol-Animal 57:104–132. doi:10.1007/s11626-020-00517-7 PMC7657088 33175307

[246] Papineni RS, Rosenthal FS. 1997. The size distribution of droplets in the exhaled breath of healthy human subjects. J Aerosol Med 10:105–116. doi:10.1089/jam.1997.10.105 10168531

[247] Chao CYH, Wan MP, Morawska L, Johnson GR, Ristovski ZD, Hargreaves M, Mengersen K, Corbett S, Li Y, Xie X, Katoshevski D. 2009. Characterization of expiration air jets and droplet size distributions immediately at the mouth opening. J Aerosol Sci 40:122–133. doi:10.1016/j.jaerosci.2008.10.003 PMC7126899 32287373

[248] Zhou J, Wei J, Choy K-T, Sia SF, Rowlands DK, Yu D, Wu C-Y, Lindsley WG, Cowling BJ, McDevitt J, Peiris M, Li Y, Yen H-L. 2018. Defining the sizes of airborne particles that mediate influenza transmission in ferrets. Proc Natl Acad Sci USA 115. doi:10.1073/pnas.1716771115 PMC5877994 29463703

